# An E-Delphi study to facilitate animal welfare assessment in Italian zoos and aquaria

**DOI:** 10.1371/journal.pone.0309760

**Published:** 2025-01-06

**Authors:** Barbara de Mori, Elena Mercugliano, Adriana F. Cerizza, Pierfrancesco Biasetti, Daniela Florio, Riccardo Da Re, Sandro Mazzariol, Olga Usachova, Simone Basile, Claudia Gili, Sara Rota Nodari

**Affiliations:** 1 Department of Comparative Biomedicine and Food Science, University of Padua, Padua, Italy; 2 Ethics Laboratory for Veterinary Medicine, Conservation and Animal Welfare, University of Padua, Padua, Italy; 3 Department of Reproduction Management, Leibniz Institute for Zoo and Wildlife Research, Berlin, Germany; 4 Department of Veterinary Medical Science, University of Bologna, Bologna, Italy; 5 ETIFOR, Padua, Italy; 6 Stazione Zoologica Anton Dohrn, Naples, Italy; 7 Italian National Reference Center for Animal Welfare, Istituto Zooprofilattico Sperimentale della Lombardia e dell’Emilia Romagna ‘Bruno Ubertini’, Brescia, Italy; National Cheng Kung University, TAIWAN

## Abstract

Ensuring animal welfare is a key aspect of animal management in zoological facilities and aquaria, representing a pivotal facet of their mission. Italy currently lacks a comprehensive and valuable assessment methodology for evaluating the welfare of captive animals. To address this gap, the present study aimed to identify the most important criteria that should be considered in the welfare management and assessment of animals housed in Italian zoos and aquaria. To pinpoint this issue, we engaged experts with diverse backgrounds, structuring their communication throughout an iterative process, by applying the Delphi methodology. A pilot and three Delphi rounds were administered online to 74 experts, asking them to a) validate relevant topics derived from current legislation and guidelines divided into three clusters: Care, Wellbeing, and Regulation; b) assess the relevance of these topics across taxonomic groups; c) propose and confirm indicators for each identified topic; d) suggest and refine a list of questions for animal welfare assessment. The results were three lists of questions, one for each cluster, with a total of 80 topics, 174 indicators, and 272 questions identified by the experts. The aspects included in these lists offer valuable insights into the main aspects experts consider relevant for captive animal welfare. Despite the complexity of animal welfare and the huge amount of species hosted in zoos limiting the possibility to cover this aspect with a single expert consultation, this project actively addresses the urgent need for standardization in animal welfare assessment, contributing to the ongoing development of zoological regulations in Italy. This is especially important given the current limited legislative framework, underscoring the link between animal welfare and successful ex-situ species conservation. These questions can be the basis of fine-tuned protocols to be tested in future projects aiming at animal welfare self-assessment, thus supporting authority inspection processes.

## Introduction

Amidst the rapid decline of biodiversity, strongly driven by human activities such as habitat destruction, pollution and climate change [1], zoological gardens and aquaria (referred to as ‘zoos’ hereafter) play an important role as conservation sites [2]. Originating between XVIII and XIX centuries from collections known as *menageries*, which primarily aimed to display native and exotic species [3], zoos have undergone a significant evolution. Nowadays, zoos are encouraged to transition into “Conservation zoos” [2] facilities actively pursuing globally integrated conservation objectives. Animal welfare has been recognized as one of the main components of their mission, alongside conservation and education [4]. Ensuring animal welfare is a *conditio sine qua non* for the sustainable management of these facilities worldwide [5]. The World Association of Zoos and Aquariums (WAZA) describes animal welfare as “(…) how the animal experiences its own world and life through its association with pleasant experiences specific for that species such as vitality, affection, safety, and excitement, or unpleasant experiences such as pain, hunger, fear, boredom, loneliness, and frustration” [6]. Zoos have a powerful function in preserving and monitoring biodiversity [7] fulfilling multiple roles, through participative educational activities, breeding and reintroduction programs of endangered species, and research initiatives that contribute to scientific knowledge about animal behaviour, adaptation, and physiology [8, 9]. Moreover, zoos offer a significant opportunity to disseminate scientific information to the general public regarding species conservation and welfare [10]. The number of visitors underscores this potential: the world’s top 1,000 zoological gardens and aquaria attract more than 600 million people annually, accounting for approximately 10% of the world’s population [11]. In zoos, this huge audience, particularly children, engages in direct, non-video-mediated eye contact with animals of diverse species. Moreover, the possibility for visitors to also interact with animals [12] must consider the physiological and ethological needs of the animals, integrating principles of animal welfare and care into public education goals [13]. To ensure these needs are met, and to facilitate methodological standardization of animal welfare assessment approaches, zoological institutions must have access to dedicated policies and guidelines, working in collaboration with competent authorities. In European zoos, animal health and welfare are regulated by various EU and national regulations. Conservation, education and welfare minimum standards are included and addressed in the European Directive 1999/22/EC [14], adopted with the aim of protecting and preserving wildlife by amplifying the role of zoos in biodiversity conservation. Since the implementation of the European directive, adopted in Italy in 2005 with the Legislative decree n. 73 [15], a total of 37 facilities have been licensed as of 2023, with an additional nine currently undergoing the assessment phase [16]. The European Directive 882/2004/CE on animal welfare has been transposed into Italian national law for domestic farmed animals for over 15 years [17]; however, there are still no guidelines specifically addressing animal welfare issues in Italian zoos, nor official shared checklists available for the competent authority to actively monitor their implementation. Therefore, the ongoing improvement of animal welfare in zoos relies merely on increasing knowledge of species’ requirements, and providing training and professional development opportunities for animal husbandry personnel [18]. Defining and assessing animal welfare in zoos is an undoubtedly complex endeavor [19]. It involves considering the diverse physiological, health-related, and ethological needs of all the different species housed, as well as the state-of-the-art housing systems, environmental enrichment techniques, staff training and the maintenance of adequate records of animal health and welfare, while at the same time ensuring accurate dissemination programs for the public [20, 21]. Given the inherent complexity of implementation, and the risk of addressing these issues without a proper scientific background, competent authorities and zoos must have access to reliable trustworthy tools, ensuring uniform and comparable inspections. Such tools would enable both, zoo staff and officers to record inspection findings systematically, facilitating the evaluation of progress over the years.

This study aimed to address this crucial gap in animal welfare assessment by facilitating an expert’s participatory consultation process. The primary goal was to develop a systematic and standardized approach for evaluating the welfare of animals housed in Italian zoological facilities by identifying the most relevant criteria to assess animal welfare. To achieve this goal, existing legislative documents were thoroughly screened, and three pivotal areas were identified, guiding the organization of the themes into three distinct clusters: Care (C), Wellbeing (W), and Regulation (R). For each cluster, we aimed to obtain a set of questions adopting a participatory bottom-up approach, involving experts in the field of animal welfare to conceive the questions.

The study was structured around the Delphi technique–a participatory approach developed in the 1950s. The Delphi method is used to facilitate a structured group communication process, in order to attain a collective opinion or decision with the highest possible level of consensus [22]. In this context, a panel of experts has engaged anonymously in multiple rounds of questionnaires, interspersed with subpanel rounds and resuming preliminary results, during which the collected responses and opinions are shared with the group in aggregated form to approach the following round. This method has been extensively used in healthcare [23], medicine [24], education [25] research [26, 27], and also in ethics [28]. Furthermore, it has been used in previous studies to identify animal welfare priorities or indicators for different species [29–31]. Notably, this study, to our knowledge, represents the first application of the Delphi method to build a comprehensive questionnaire to assess animal welfare in Italian zoos, aiming to reach the highest possible level of consensus on the parameters and evaluation methods. The results here provided comprise three draft lists of questions (one for each priority cluster) obtained throughout the Delphi consultation. These three lists of questions could be the basis of fine-tuned protocols to empower facilities to conduct self-assessment activities and provide the competent authorities with the means to perform consistent inspections and audits, ultimately aiming to elevate management and welfare standards. A future project will then entail the review and further refinement of the list of questions, featuring an applicative testing process conducted within zoological facilities and aquaria. Once validated through testing, the questionnaire could then be utilized to create a practical toolkit, benefiting not only Italian facilities but also extending its utility to European countries that have yet to comprehensively address, regulate, and harmonize animal wellbeing management within their respective zoos.

## Materials and methods

### Delphi methods

This study employed the Delphi technique, a method for structured group communication developed to elicit and prioritize expert opinion when dealing with limited or non-existent information, and to obtain new insights on current or prospective issues [22]. It operates as an iterative technique, using sequential questionnaires and group feedback while ensuring participant anonymity during the process [28, 32]. Traditionally, a Delphi study involves an initial round devoted to information retrieval, followed by prioritization rounds until consensus is reached [33]. However, the Delphi method is highly adaptable, allowing for variations such as starting with literature reviews to inform the first round, and incorporating discussion boards [34].

In the present study, a pilot trial and three Delphi rounds were conducted through the online survey software LimeSurvey [35], upholding participant anonymity while supporting response tracking.

### Ethical statement

The study was performed in compliance with the relevant ethical and normative guidelines of Europe and Italy. No approval of an ethics committee/institutional board was needed at the time of the study since participants voluntarily agreed to participate before the beginning of the study. Participants were assured of anonymity, and their participation could be canceled at any time without any justifications. A privacy notice and written informed consent were provided to inform and assure anonymity and confidentiality, informing participants that the information and data collected would be used for research purposes only and analyzed in an aggregate way.

### Panel composition

The Delphi study panel comprised individuals deemed ‘experts’ within the specific field related to the study subject [21, 25]. The panel experts were recruited through a non-discriminative exponential snowball sampling process from 27 July 2021 to 15 October 2021 [36], commencing with network contacts within the Department of Compared Biomedicine and Food Science of the University of Padua and IZSLER.

Before approaching potential panelists for this Delphi study, we established selection criteria emphasizing the need for diverse backgrounds among participants, to mitigate potential biases. Knowledge and expertise in zoo management, animal welfare research and assessment, animal care, and biodiversity conservation research were considered. Invitation emails were extended to identified participants, including a brief study overview, the Delphi information sheet, and contact details [28]. Prospective experts were then prompted to confirm their participation and provided with contacts of the lead research team in case of inquiries. Experts initially engaged from operational units were then individually asked to identify and suggest additional participants, following the snowball sampling approach.

Participation in each of the following Delphi rounds was confirmed or denied individually by each participant through email communication. A total of n = 74 Italian experts responded to the initial call, engaging anonymously in one or more clusters of the questionnaires (Care, Wellbeing, Regulation) according to their expertise. The sex ratio of the expert participants was skewed in favor of males (59,45%) versus female experts (40,54%). Notably, all the initially recruited experts effectively participated in at least one round. Information regarding recruited participants (name, position, and contact details) was collected adhering to current European privacy regulations [37] and saved in an Excel file.

### Data collection and analysis

After a preliminary review of the relevant legislative sources, best practices, and guidelines to identify potential themes and associated topics to be included in the questionnaires, four E-Delphi survey rounds (a pilot trial and 3 Delphi rounds) were performed among an invited panel of experts. S1 Table summarizes the definitions of relevant terms used in the present study (e.g. theme, topic, etc.).

To review existing legislation and guidelines in Europe on animal welfare assessment in zoos, a targeted web search was performed using keywords such as "animal welfare guidelines", "animal care guidelines", "zoos and aquaria" and "zoos and aquaria legislation". Based on the results, European guidelines were obtained and analyzed to identify the most suitable ones for the Italian context. The selected guidelines, norms, and EU and Italian legislation are presented in Table 1. This helped to identify important themes and topics related to the welfare of animals in Italian zoos, which were used to inform the content of the first round of the Delphi study. We have then searched these topics within the current Italian zoos legislation [15].

**Table 1:** List of the documents used for the selection of the themes and topics for the pilot Round.

Document title
Italian Legislative Decree No. 73 of 21 March 2005 [15]
Council Directive 92/65/EEC of 13 July 1992 (Balai) [38]
Convention on International Trade in Endangered Species of Wild Fauna and Flora (CITES) [39]
Gutachten über Mindestanforderungen an die Haltung von Säugetieren [40]
EU Zoos Directive Good Practices [14]
Zoos Expert Committee Handbook [41]
Caring For Wildlife The World Zoo and Aquarium Animal Welfare Strategy [20]
Welfare Quality Principles and Criteria of Good Farm Animal Welfare [42]
Five Domains Model [43]
European Association of Zoos and Aquaria (EAZA) Standards for the Accommodation and Care of Animals in Zoos and Aquaria [44]
EAZA Guidelines on the use of animals in public demonstrations [45]
WAZA Guidelines for the use of animals in visitor interactions [13]

The pilot and 3 consecutive survey rounds of the E-Delphi study were conducted for each designated cluster (Care, Wellbeing, Regulation) and presented in Italian language to ensure full comprehension of the texts and contents for all the participants. Ahead of the pilot round, international experts were engaged to evaluate the content and the appropriateness of the approach, with a specific emphasis on the relevance of the proposed topics for each theme to be included in the E-Delphi questionnaire. Once feasibility had been confirmed through the pilot phase, an email containing a link to the first round of the E-Delphi was sent out to the Italian experts who had agreed to participate. Each round featured a project description, guidelines on how to complete the questionnaire, contact information of the lead research team, and a mandatory informed consent form. The duration for each round was one month, and reminder emails were sent to enhance the response rate [46].

**Fig 1:**
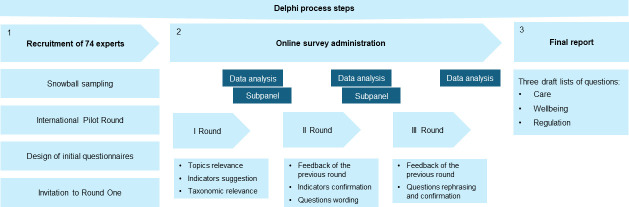
Flow diagram of the Delphi process.

Between E-Delphi rounds, a dedicated subpanel, comprising a smaller group of experts (n = 12), offered input for the refinement of the ensuing rounds. The subpanel provided additional inputs to refine the process, finalized questionnaires and feedback, and reduced the redundancy of answers [47–49]. Moreover, it was useful for organizing the diverse and varied nature of the information collected during the subsequent rounds, derived from multiple sources and varied widely in the formulation. Furthermore, the subpanel was useful for evaluating those individual themes for which a low consensus threshold and a high amount of different opinions emerged during the process. A flow diagram of the Delphi process is shown in Fig 1.

The Round One questionnaire, distributed to all the participants, encompassed topics and themes (some examples are reported in Table 2) identified through legislation consultation, the Pilot Round, and subsequent evaluations.

**Table 2:** List of examples for the themes addressed by Round One.

Cluster	Description	Themes
Care	Physiological needs of animals, veterinary care and training	• Enclosure configuration • Environmental parameters of enclosures • Mixed Exhibit • Management • Welfare and reproduction • Animal Training
Wellbeing	Relations between animals and visitors, and other aspects of animal welfare not addressed by the cluster Care and Regulation	• Animal group formation • Environmental enrichment • Psychological needs of housed animals • Interaction with the keeper • Interactions with visitors • Management • Training of animal care staff
Regulation	Registry, staff and animal handling	• Animal management • Cleaning, Disinfection & Hygiene • Quarantine—isolation • Infectious diseases and zoonosis • Post Mortem • Identification—Registration • Conservation and research projects • Training of personnel dealing with animals

Participants were asked to 1) confirm the relevance only for those topics that are not already present in the current legislation; 2) suggest the most suitable indicators (at least one for each topic) and/or evaluation methods for assessing the topics considered to be relevant, or that are already legal requirements in Italy; 3) indicate whether a topic applies to a specific taxonomic group or could be applied or extended to all the taxa proposed by classes (mammals, birds, reptiles, amphibians, fish and invertebrates), considering the specificity that the Italian law already includes unique criteria only for a single mammal species, bottlenose dolphins (*Tursiops truncatus*). Participants were presented with options based on the Pilot Round outcomes and invited to review and/or amend their evaluation. To further enhance expert input and elucidate their choices and ratings, participants were given the opportunity to add free-text comments. Data collected were anonymously inserted into a spreadsheet assigning a code to each topic (Cr1, Cr2, etc. for Care, Wb1, Wb2, etc. for Wellbeing, Nv1, Nv2, etc. for Regulation). Concerning relevance, a topic achieved relevance status if it reached a consensus threshold of ≥90% among participants. Since in Round One experts should indicate if each topic was equally or differently relevant for all taxa, or not relevant at all, relevance was calculated as the sum of experts that indicated a topic to be equally relevant for all taxa and those who indicated that the topic was relevant but with marked differences among taxa. Percentages were calculated on the number of experts who replied to the questionnaires (excluding the experts who did not reply to the cluster questionnaire and the specific question). At the end of Round One, the subpanel was involved to review and refine the information collected.

The Round Two questionnaire, derived from the revision of Round One outputs by the subpanel, was sent to all participants divided into three clusters (Care, Wellbeing, Regulation). In this round, panel members were asked to: 1) confirm the formulation of the indicators suggested in Round One and 2) propose the “wording and terminology” for indicator-based questions pertaining to the measurement of individual topics. Indicators were maintained to be proposed in Round Three when the majority of respondents (>50%) confirmed their relevance; data was exported from the online survey platform in a dedicated spreadsheet file to calculate the number of replies confirming the relevance of each indicator. To be consistent with the methodology and validate the process, the subpanel was again engaged after Round Two to revise the outputs.

In Round Three, the goal was to ask participants to confirm the list of proposed questions for each indicator and offer suggestions for potential rephrasing. Additionally, participants were asked to confirm which questions for each indicator within each topic they wished to retain. Questions and indicators were maintained when the majority (>50% of experts) confirmed their relevance. The outcome of Round Three was a set of questions for each cluster further revised at the practical level by the subpanel. To further exemplify the results of this final round and their significance for the different aspects related to animal welfare in zoos, we divided the questions of the final lists into macro-categories obtained starting from the themes presented in Round One. In addition, we created word clouds, one for each cluster, to visually illustrate the key concepts around which the three lists of questions articulate.

## Results and discussion

By comparing the topics identified to create the questionnaires with the current Italian legislation [15], we highlighted that 27 are not yet covered or implied, such as “Substrate or flooring suited to the needs and characteristics of the hosted species” (Cr7); “Corrective and preventive actions taken in case of zoonosis” (Nv37); “Preliminary evaluation before acquiring new specimen” (Wb11). S2 Table summarizes the topics that are not specifically included in the current Italian legislation. Of the 74 Italian experts who responded to the initial call, some participated in multiple clusters as follows: 7 in Care and Wellbeing, 1 in Care and Regulation, 1 in Regulation and Wellbeing, and 11 in all three clusters.

### Round One results

The results of the Pilot Round, upon review, generated 23 topics for Care, 1 for Wellbeing, and 26 for Regulation to be evaluated and integrated in Round One. Round One questionnaire received 91 overall responses, distributed as follows: 30 for Care, 30 for Wellbeing, and 31 for Regulation, with a response rate of 85.8%. Experts were given the possibility to add comments to explain and specify their answers. In the three clusters, the topics proposed in Round One reached a consensus threshold of 90% and thus were considered relevant by experts as follows: for Care cluster, 27 topics (54%) of 50 proposed; in Wellbeing, 10 topics (25%) of 40 proposed; in Regulation 35 (81.39%) of 43 proposed.

Regarding the application of topics to different taxa, the topics concerning aspects already included in the current legislation were considered valid and applicable to all the taxa; therefore, experts were not asked to specify the relevance of these topics for taxonomic groups, with the exception of aspects focused only on bottlenose dolphins in the Italian legislation (1 topic for Wellbeing, 2 for Regulation, and 3 for Care). Indeed, since these aspects are specified only for one species, we deemed it necessary to verify if they should be applied to other taxonomic groups. For all the other topics generated during Round One, experts had to indicate if a topic should be included in the final list of questions exclusively for one or more specific taxonomic groups, instead of all. Concerning the specificity of topics, the consensus threshold of 90% set for this round was not reached for any of the topics, indicating a prevailing opinion among experts to maintain the generality of these topics without exclusivity to specific taxonomic groups in the list of questions. Following these results, we opted not to categorize the topics by taxonomic groups, choosing to report here only the ones that reached a consensus threshold of at least 50%. In the Regulation cluster, only the topic “Identification of individuals” (Nv41) was considered relevant for different groups with a majority of consensus (51.7%). In Care, the topic “Adequate animal training (Frequency and duration of sessions, type of positive reinforcement use)” (Cr65) obtained a consensus of 74%. The topic “Possibility for animals to be exposed to direct solar light” (Cr15) was considered relevant for different taxa, endorsed by 50% of participants. Conversely, in Wellbeing, no item was considered relevant for different taxa by 50% or more of the participants.

In Round One, experts were also required to put forth at least one indicator for each topic. Outcomes included 145 indicators for Care, 25 for Wellbeing, and 206 for Regulation. An example of a specific topic and suggested indicators for each cluster (Care, Wellbeing, and Regulation) is shown in Table 3.

**Table 3:** Examples of themes and suggested indicators for a specific topic for each cluster.

Themes and examples of topics and indicators	
Care	
Example of a chosen theme	Management of the housed animals
Topic of example related to the theme	Cr1—Presence of suitable premises or facilities for injured, sick or stressed animals
Suggested indicators for the topic	species-specific enclosure sizes in accordance with EAZA guidelines (where available or other scientific literature); in the absence of specific guidelines, evaluation by the Curator and/or the structure veterinarian presence of facilities dedicated to animal treatment; presence of a sheltered area protected from weather and atmospheric agents; access is restricted to dedicated staff and veterinarians only.
Wellbeing	
Example of a chosen theme	Creation of animal groups
Topic of example related to the theme	Wb1—Preliminary assessment before acquiring a new species/individual
Suggested indicators for the topic	species-specific enclosure sizes in accordance with EAZA guidelines (where available or other scientific literature); in absence of specific guidelines, evaluation by the curator and/or the structure veterinarian Presence of a plan for introducing new species safely into an exhibit.
Regulation	
Example of a chosen theme	Housed animals’ management
Topic of example related to the theme	Nv2—Daily monitoring of animal health
Suggested indicators for the topic	presence of daily keeper records; presence of trained keepers in sufficient numbers; examination of health status and, where applicable, behaviour by carrying out regular and random visual inspections; nutrition and hydration status assessment; regular food and water intake monitoring; body weight monitoring; major organic functions control; presence of normal faeces according to species; assessment of the animal’s interest in its environment periodic evaluation of ethograms; presence of behavioural anomalies; sensorium state evaluation (normal reactivity to natural or provoked stimuli); veterinary intervention in case of abnormalities/alterations; presence of clinical records; presence of specific protocols and checklists; daily number of checks recording.

### Round Two results

The topics and indicators obtained in Round One and revised by the subpanel were presented to the participants at the beginning of Round Two. Some examples of topics that were excluded from Round One to Round Two because they did not reach 90% of consensus threshold are listed in Table 4.

**Table 4:** Examples of topics that did not reach consensus from Round Two to Round Three.

Cluster	Topic
**Wellbeing**	Wb6—Impact evaluation on the social group before and after removal of one of its individuals
**Care**	Cr5—Design of exhibits and tanks that consider the species-specific needs of the animals housed, giving them the possibility to use all available space (length, width, depth/height)

Here we report two examples of topics excluded by the subpanel and thus not proposed in Round Two: for Wellbeing “Wb17—Opportunities for animals to solve problems that lead to positive experiences (rewarding challenge)”; and for Regulation “Nv32—Proper duration and management of animals in isolation/quarantine”.

In this round, experts were asked to confirm the indicators of Round One and formulate questions based on the suggested indicators, which were previously reviewed and integrated by the subpanel. We received 33 replies for Care, 29 for Wellbeing, and 31 for Regulation, with a response rate of 87.73%. For the Care cluster, 157 indicators were proposed to the experts in this round: the 12 indicators that exceeded the original 145 generated in the previous round by experts were suggested by the subpanel. Among these, 152 indicators were confirmed by the experts, 1 was excluded, and 4 received the same number of responses in favor of confirmation and elimination. All the 157 indicators reached the following round as they were considered relevant by the subpanel. For the Wellbeing cluster, 24 indicators of the 25 proposed in this round were confirmed by all the experts, as well as all the 206 indicators regarding Regulation. In this round, a total of 1,117 possible questions based on the indicators were proposed by experts: 412 for Care; 93 for Wellbeing, and 612 for Regulation. To maintain consistency throughout the process, the outputs of Round Two were revised by the subpanel before submitting them to Round Three. Table 5 provides examples of the suggested questions for each category after their phrasing and formulation were refined during Round Three.

**Table 5:** Examples of questions suggested by experts for an indicator per cluster in Round Three.

Examples of questions suggested for the corresponding indicators	
Care	
Topic	Cr10—Adequacy of shelters for adverse weather conditions in external enclosures
Indicator	Adequate maintenance of temperature and humidity inside the shelters and possible control of the same through adequate equipment
Suggested questions	For the species with the specific necessity of temperature and humidity, are they respected monitoring them through specific equipment?
	Is there adequate equipment for monitoring the temperature and humidity of enclosures and tanks?
Wellbeing	
Topic	Wb 29—Opportunities for animals to stop interactions with visitors and move away
Indicator	Indications of behavioural rules to be observed by visitors
Suggested questions	Is there a protocol to describe the behavioural rules to be observed by visitors in accordance with EAZA guidelines and/or equivalent and/or scientific literature and/or curator/veterinarians’ indications?
	Is there a methodology to monitor the respect of the protocol?
	Are there signs and indications to inform about visitors’ norms of behaviour?
Regulation	
Topic	Nv7—Regular implementation of preventive medical measures
Indicator	Evidence of the presence of a preventive medicine plan, including vaccinations against diseases of the species
Suggested questions	Are prophylaxis and disease prevention planned?
	Is there an annual animal disease control plan available?

### Round Three results

In Round Three, 27 answers were received for Care, 19 for Wellbeing, and 33 for Regulation, yielding a response rate of 74.52%. Questions that conveyed the same meaning but showed different wording were maintained, indicating all the indicators and topics they would match. Some rewording of questions was employed, to integrate experts’ suggestions, enhance linguistic syntax and ensure overall clarity. This harmonization of wording and sentence construction was indeed necessary to guarantee consistency across the draft. Table 5 shows an example of the questions proposed for one indicator for each of the three clusters to be included in the list of questions.

In summary, the Delphi results contain topics, indicators, and corresponding questions for each cluster as shown in Table 6.

**Table 6:** Structure of the results of Round Three.

Clusters	Care	Wellbeing	Regulation
**Topics**	35	7	38
**Indicators**	66	13	95
**Questions**	84	18	170

**Fig 2:**
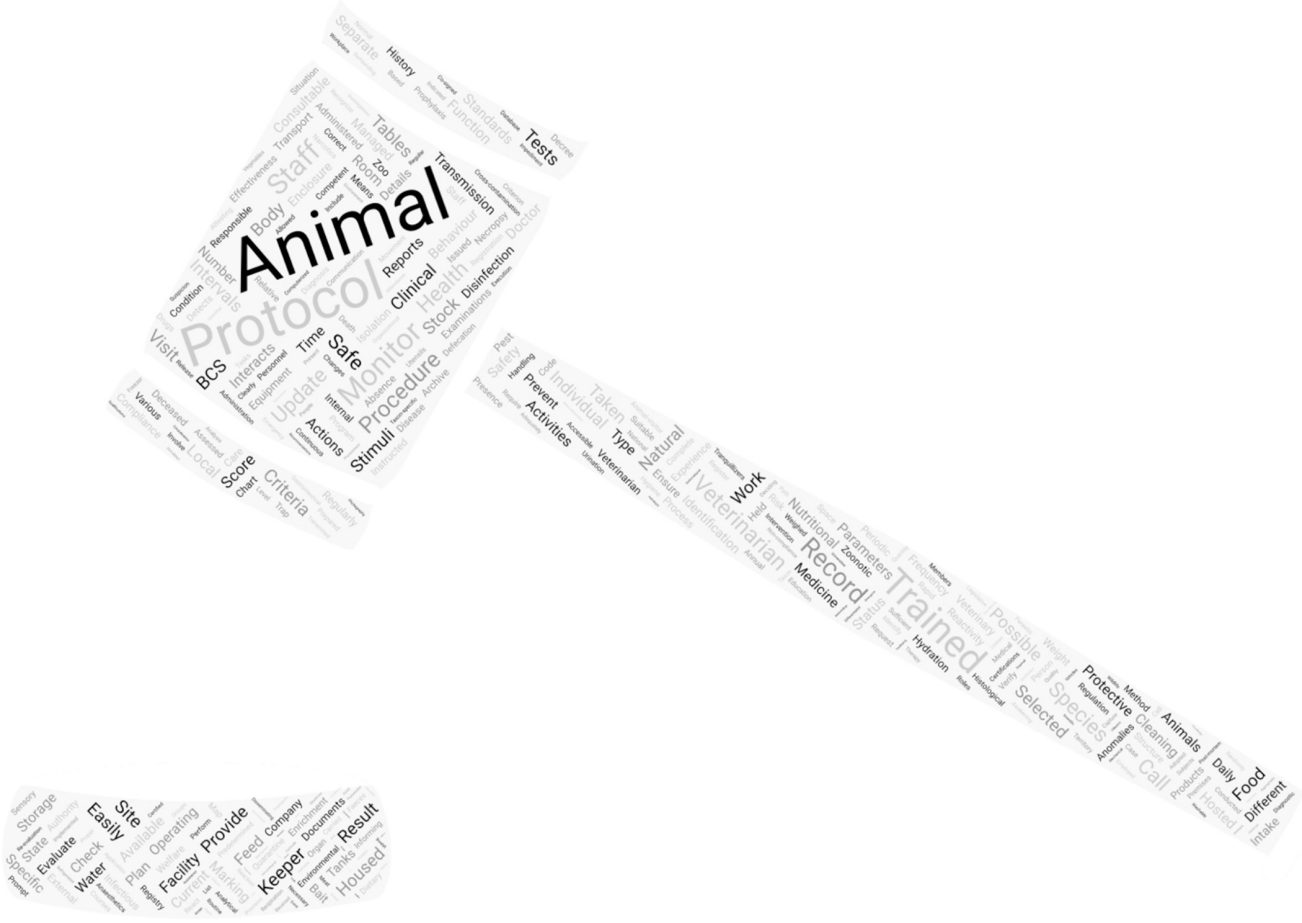
Structure of the lists of questions with the number of topics, indicators, and questions.

The structure of the three draft list of questions is graphically shown in Fig 2.

Throughout the process, 6 topics for Wellbeing, 13 for Care, and 15 for Regulation were consistently maintained from the Pilot Round until the end of Round Three. Following the results of Round One concerning relevance for taxonomic groups, questions were not divided by taxa, as they apply to all species. Here, we outline the predominant topics (containing the highest number of questions) per cluster. In the Care cluster, the topic “Appropriate safety of enclosures and tanks in relation to housed animals” (Cr20) features prominently, presenting 6 indicators and 7 related questions, in line with national safety standards as well as animal welfare regulation. This topic contains indicators concerning, for example, the application of EAZA guidelines, sanitation protocols, safety measures (e.g. presence of locks, padlocks, and barriers), and protocols to prevent animal escape. For Wellbeing, the topic “Preliminary evaluation before acquiring new individuals” (Wb2) received the highest number of indicators (n = 5) and 7 questions. In this topic, some of the indicators are focused on the periodic census of animals, the presence of a quarantine area, and the evaluation of compatibility with the preexisting group of animals in which the new individual will be included. In the Regulation cluster, the topic that contains the highest number of indicators and questions, respectively 13 and 33, was “Monitoring the health conditions of the animals according to their species” (Nv2). This topic covers a wide array of subjects, including the assessment of nutrition and hydration states, veterinary intervention, and regular monitoring of animals’ weight and behaviour.

**Fig 3:**
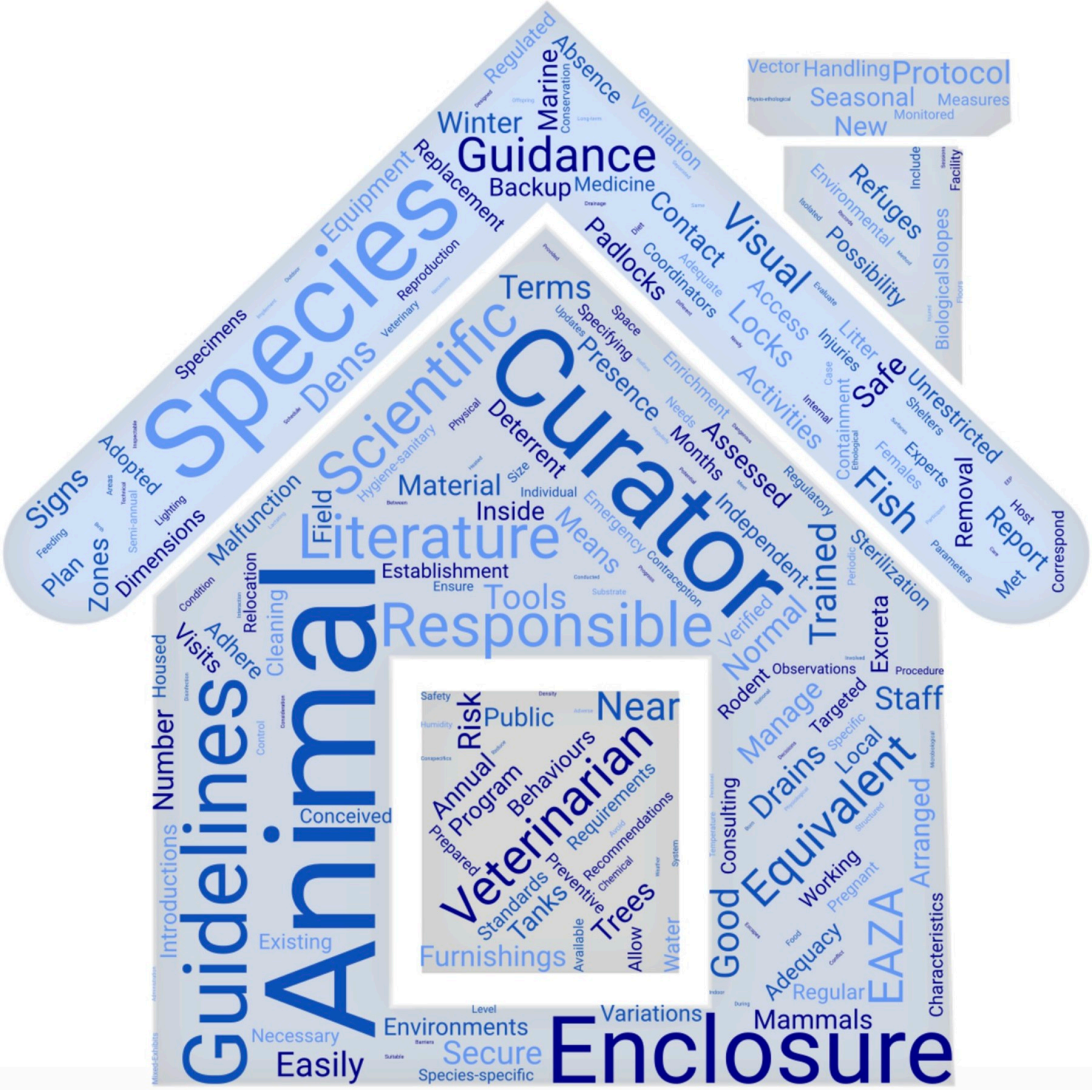
Spider web showing questions distribution into the macro-categories for the three cluster lists.

To provide further insights into the aspects related to animal welfare in zoos covered by the three lists, the questions were divided into macro-categories (Table 7) obtained starting from the themes proposed in Round One. Fig 3 shows how questions of the three clusters are distributed in the macro-categories.

**Table 7:** Structure of the results of Round Three.

Macro-categories	Care	Regulation	Wellbeing
Health management and veterinary medicine	0	64	0
Staff training	0	29	0
Nutrition	1	14	0
Safety	0	16	0
Behaviour and enrichment	0	10	2
Regulation	0	9	7
Husbandry	30	27	0
Housing	29	0	0
Reproduction	10	0	0
Population management	4	0	3
Animal training	9	1	0
Conservation and research	1	0	0
Visitor interaction	0	0	6
**Total number of questions**	**84**	**170**	**18**

**Fig 4:**
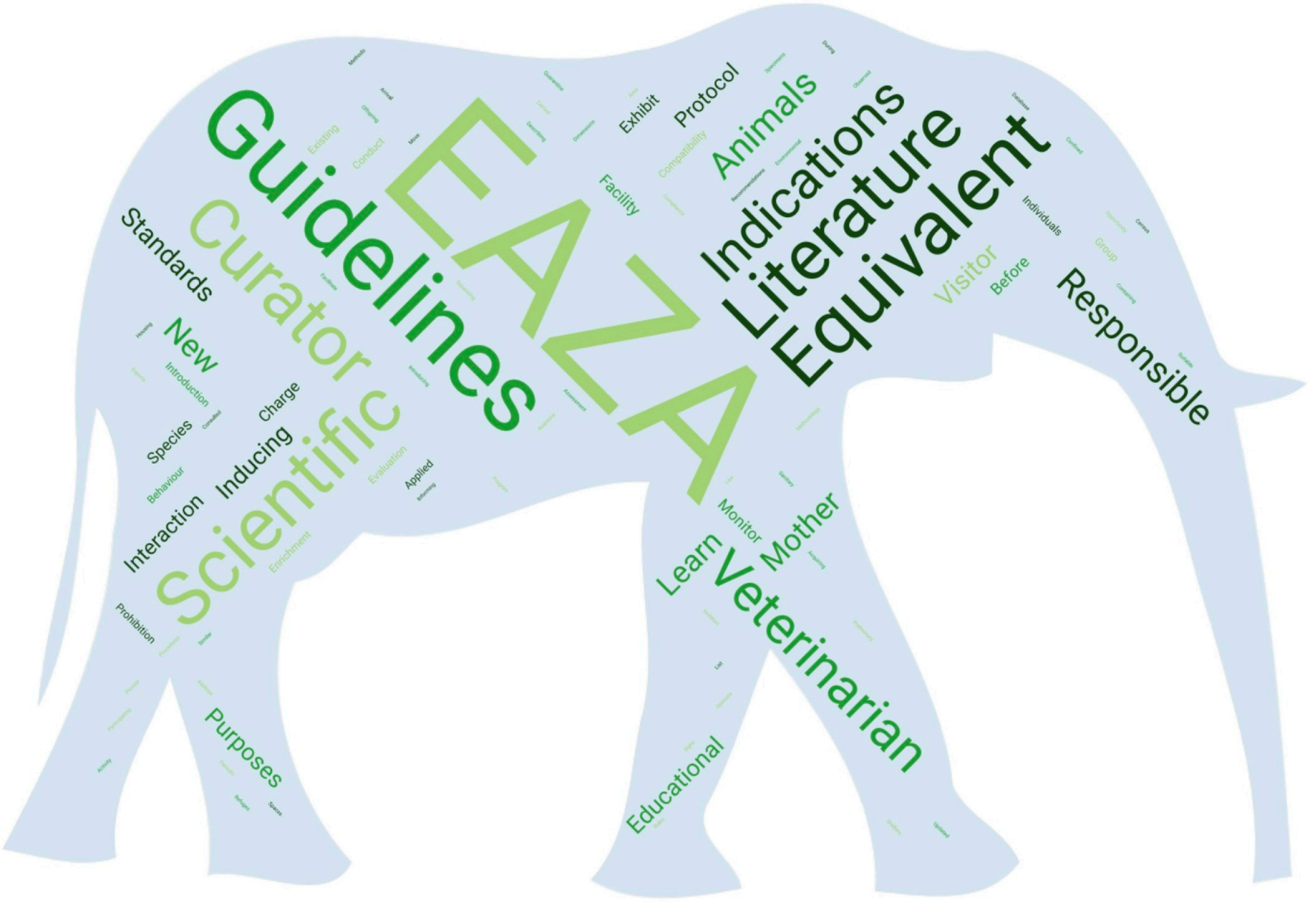
Wellbeing word cloud.

**Fig 5:**
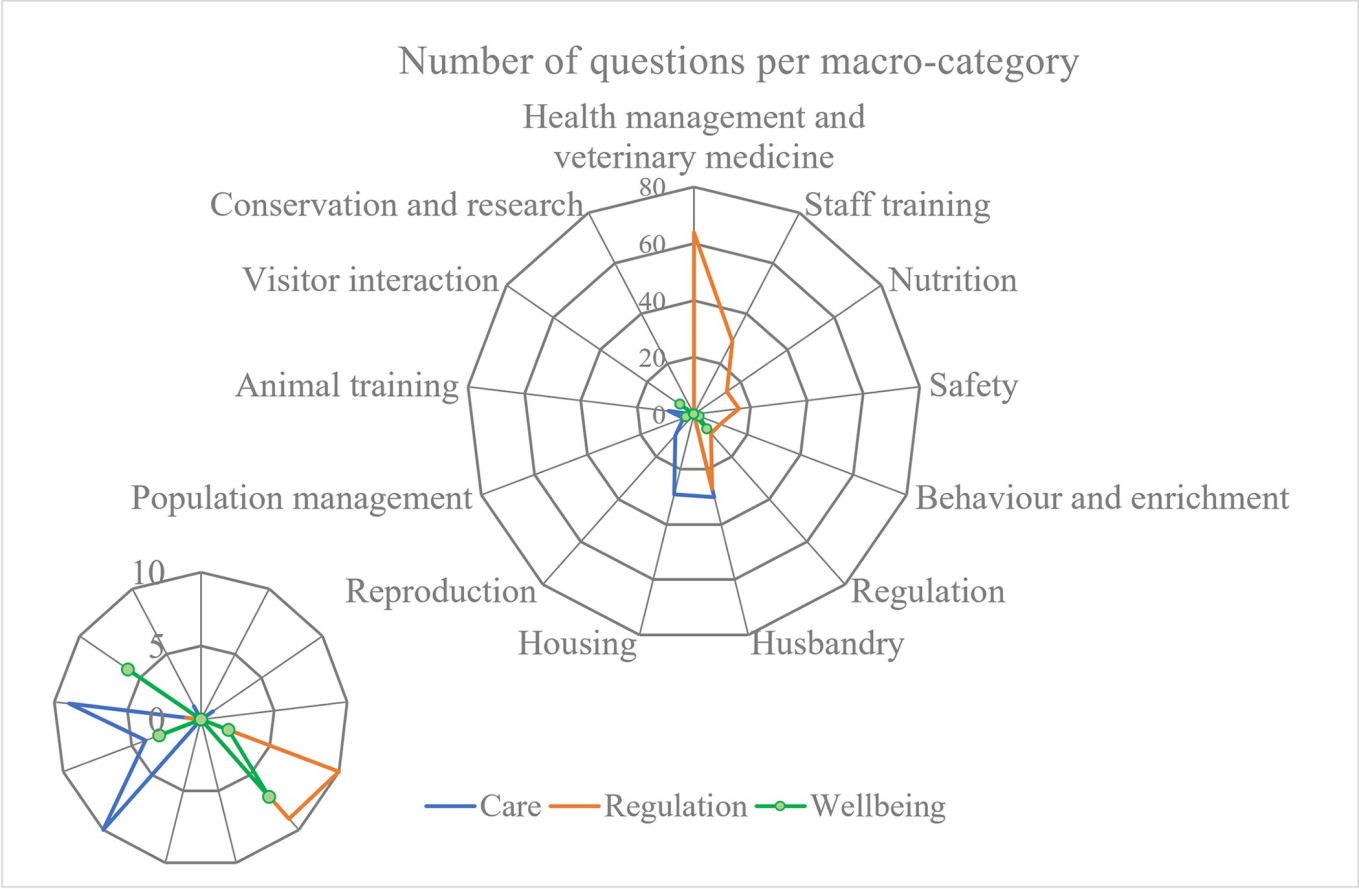
Care word cloud.

**Fig 6:**
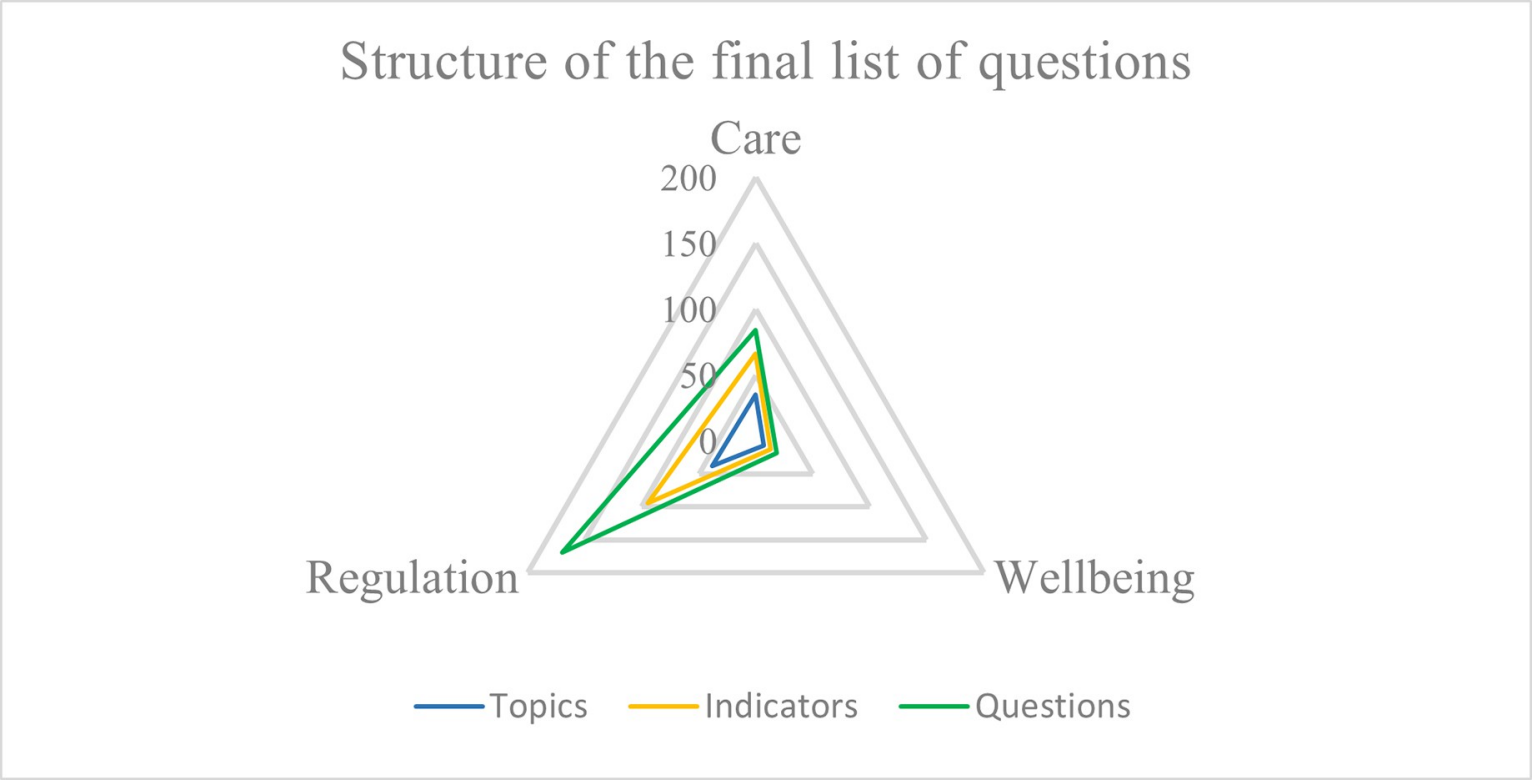
Regulation word cloud.

In addition, we created a word cloud for each cluster to visually represent Wellbeing with an elephant (Fig 4), Care with a house (Fig 5), and Regulation with a gavel (Fig 6) and highlight the key concepts around which the three lists of questions are articulated.

S3–S5 Tables contain the list of questions resulting from Round Three (the list is provided in English language, but the original one is in Italian). The list represents the draft that could be used as a basis for future projects that aim to design a provisional toolkit to be used during the following testing and refinement, together with the current literature available.

## Discussion

To our knowledge, this is the first study conducted in Italy aimed at the development of a questionnaire specific for the assessment of animal welfare in Italian zoos. The Delphi technique was used to build a first draft list of questions through experts consultation. This methodology aims to reach a consensus on a specific issue and was previously used in other studies for the assessment of the welfare of domestic and laboratory species [32, 33, 50]. The robust engagement of experts, indicated by consistently high response rates across rounds and the vast number of suggested topics and indicators, underscores the relevance of the subject, as well as the stability and appropriateness of proposed topics for the study. However, it is noteworthy that some participants changed their view on some topics’ relevance between the Pilot and Round One; this highlights the importance of a testing phase to set up procedural guidelines, and aligns with a key aspect of the Delphi method, which is the opportunity for participants to change their opinions [28, 51]. This shift of opinions could have been influenced by participants consulting Pilot results before Round One, possibly swaying minority opinions toward the majority’s position [52].

Among the topics proposed in Round One, 27 were deemed not to be present in the Italian current legislation dedicated to zoos [15]. These items are included in the three clusters, looking at animal management from different perspectives, that cover housing, behavioural and training management, risk assessment, and staff training. Regardless of being present in the final results with the different kinds of questions among clusters, these items certainly deserve attention at the legislative level as well as for daily application of animal care procedures.

The higher number of topics confirmed for the cluster Regulation in the Pilot and the first round can be due to the necessity of zoos to know and act in compliance with current national laws (i.e. “Individual identification” and “Census record of hosted animals”). The high relevance maintained between Pilot Round and Round One in Wellbeing for topics relative to infectious diseases and zoonosis could be due to the adaptations required in zoos during the SARS-CoV-2 pandemic and possible virus transmission to animals hosted in zoos [53, 54]. Experts’ change of opinions can be due to their belonging to different fields. However, the stability of opinions can be also linked to individual variability and behaviour [55].

The results of Round One were presented to a subpanel in the different clusters to confirm which thematic would have reached Round Two. To be consistent with this method, the subpanel was also consulted at the end of Round Two to decide which topics would be included in Round Three. Involving a subpanel from the beginning can be useful to revise the questions [47–49], potentially increasing the response rate.

Despite the diverse backgrounds and fields of expertise of the participating experts, Round One’s outcomes reveal an agreement to apply all proposed indicators across taxonomic groups. This consensus confirms that indicators and topics were comprehensive and exhaustive, ensuring to address the diverse needs and requirements of different species. Designing a questionnaire with broadly applicable indicators and questions enhances its practicality, thus facilitating standardized welfare evaluations, especially in facilities housing a diverse range of species, such as zoos and aquaria. Our results suggest that the aspects relevant to animal welfare should be applied to all taxonomic groups. Conversely, in Italian legislation only one mammal species (bottlenose dolphin) receives special attention with dedicated paragraphs that cover most of the indicators highlighted in this study, somehow jeopardizing the attention given in such detail to other species. Sending and reaching decision-makers and zoo and aquaria practitioners with the message that these aspects should be equally applied to all species, giving them the same dignity and importance, could be a significant achievement of this paper.

Among the animal-based welfare aspects advancing through the process up to Round Three, the necessity to provide animals with environments that allow them to engage in species-specific behaviors emerges as a crucial criterion, consistently applied to address and assess animal welfare [30, 56, 57]. Two topics of the list obtained after Round Three, both part of the cluster Care, explicitly focus on species-specific behavior, particularly concerning enclosure complexity and materials. The associated indicators and questions emphasize adherence to EAZA guidelines or reliance on current literature or veterinarian recommendations for species without established guidelines. This emphasis aligns with the presence of EAZA members’ staff in the panel, who are aware of existing guidelines to provide animals with an environment that stimulates natural species-specific behaviours. The interaction with visitors is a matter of concern for both human and animal safety, as well as overall animal welfare [9], becoming the focus of many studies and confirming the urgent need to include this issue in a pragmatic animal welfare assessment [12, 58–61]. The alignment between scientific literature and the three Wellbeing topics (comprising 7 questions) in the final list indicates that experts identified current relevant aspects and needs in animal welfare, affirming the appropriateness of the Delphi method. In addition, the highest number of indicators and questions within the Wellbeing cluster concerned the evaluation of various aspects before welcoming new individuals, including considerations of compatibility with the preexisting group. This aspect is of paramount importance for animal welfare, since the introduction of a new individual may trigger situations of aggressiveness and hostility, affecting social integration [e.g. 62, 63]. For species involved in breeding programs, this consideration becomes even more critical for conservation purposes [64–66]. Another aspect included in this topic is the census of animals hosted by a facility, however this is required by law. However, the Wellbeing cluster features a lower number of topics and indicators that reached Round Three, compared with the other two categories. Opportunities for enhancement can be explored in future projects, involving stakeholders (zoos) in direct testing of the individual questions in a self-assessment system. This application might provide valuable feedback to further suggest additional aspects to be included in the inspection official protocols.

In the Care cluster, 35 topics, 66 indicators, and 84 questions were included in the draft list of questions. The majority of indicators and questions of the draft list obtained for this cluster is concentrated on the topic relative to enclosure security, possibly because facilities have to comply with legislation on this matter as part of their daily management practices, and thus experts are more inclined to highlight specific indicators and issues.

For the Regulation cluster, the draft list incorporates 38 topics, 95 indicators, and 170 questions. The majority of indicators related to the evaluation of animals’ health based on species, indicating the high emphasis of experts on this matter, aligning with established norms and regulations.

During the identification of macro-categories created to merge the final questions, we cannot ignore the lack of questions related to conservation and research projects, which is relevant, if not mandatory, for any zoo [20]. In addition, this aspect is included in the objectives of the Italian law for zoo licensing [15]. This item only appeared once in the Care cluster with the question “Does the facility participate in species conservation programs at local, national, and/or international levels?” (Cr64). Being such a general omni-comprehensive question, its single presence could somehow be sufficient as long as the utilizer of the lists of questions for self-evaluation and inspections addresses it in detail.

The Delphi method with expert consultation demonstrated its applicability when building an animal welfare list of possible items tailored for zoos. The results of this study paved the way for the inclusion of welfare aspects and issues endorsed by expert opinions into the list of questions. The insights gathered from experts’ consultations allowed us to build three draft separate lists of questions, one for each cluster, including the items proposed by experts in Round Two and consequently formulated in Round Three.

The future development of this consensus process based on a high level of experience in other similar projects might allow the creation of an easy-to-use and science-based practical tool, to be also applied by the competent authority for a unanimous comprehensive assessment process of animal welfare in zoological facilities. The three lists of questions aim to establish standardized principles and controls, allowing for the systematic record-keeping possibility of the different assessments over the years. Finally, through its specific focus, this list of questions can be further implemented by individual facilities at all levels of organization (both top-down and bottom-up), to encourage and support animal welfare improvement in Italian zoos.

### Limitations of the study and future developments

This study presents some limitations and challenges that require further attention. The first is related to the complex, multi-faceted nature of animal welfare and its assessment, also reflected by the various existing definitions [12] and underscored by the fact that very limited (if none) checklists are currently available worldwide on this matter. Attempting to encapsulate this complexity within a general monitoring tool is an inevitable simplification, and this aspect is further complicated by the presence of vastly different taxa hosted in zoos and aquaria. It is indeed challenging to cover this aspect within a single expert consultation.

Zoos and aquaria host hundreds of species of different taxa, ranging from invertebrates to mammals, within confined spaces managed by humans and where the settings can be very diverse among facilities, change over time and have different purposes: education, conservation, research and exhibit only [67]. The chosen focus on the three clusters of Care, Wellbeing, and Regulation in this study, while providing an organized process, proved to be very complex to analyze and discriminate between each taxonomic group, particularly because some taxa notoriously receive more attention from zoo organizations, the scientific community, and the national legislation compared to others [15, 68]. Notably, 82% of animal welfare studies are focused on vertebrates, 75% on mammals and, in particular, on great apes [69], leaving taxa with lower information availability at a disadvantage: it could be difficult also for experts to give a uniform opinion about animal welfare issues based on scientific evidence, to be included in a predisposed standard toolkit. On the other hand, for species with a huge amount of information available, experts might react by declaring that “it is impossible to include all different measurable aspects within a single tool” which is already very vast, and therefore the answers might become so general that in fact could be seen as meaningless.

This study also sheds light on topics that were deemed “not relevant” by experts and that were therefore excluded from one of the rounds during the process. For instance, “Fences and barriers of the wards well related to the characteristics of the species housed” or “Adequate cleaning of the tanks”. These aspects might be relevant for specific taxa, suggesting that the diverse backgrounds of the experts (e.g. species-specific expertise, stakeholder group affiliation, facility, position, etc.) could have influenced and led to the rejection of these topics. Therefore, to foster a more balanced and fair process, it would be crucial to have a balanced number of experts with similar backgrounds and belonging to the same stakeholder category; this precaution would prevent individual views from disproportionately driving topic exclusion or inclusion [70, 71]. Therefore, we suggest including an analysis of interests as a preliminary step of a Delphi study, especially if focused on animal welfare, in order to harmonize the composition of the final expert panel.

Engagement is a fundamental aspect of the Delphi method, yet it could also represent a limitation, highlighting the imperative need for a highly motivated panel [28]. Despite the efforts taken to minimize drop-out, the response rate experienced a decline across the three rounds. However, free text comments received in each round shed light on the remarkable engagement level of those participants who completed all three rounds. Aligning with current literature, we underline the importance of effective communication, particularly during the initial phase of participant involvement. This communication must be timely, detailed, and clear enough to enable participants to grasp the commitment required, in order to either participate consciously or to respectfully decline. Such clarity ensures that the results obtained are reproducible and valid. The E-Delphi could have facilitated communication with experts, allowing us to program and maintain a repetitive email contact thus keeping an open reminder channel. Furthermore, the online approach allowed us to monitor the progress through each questionnaire, using the LimeSurvey online software, facilitating a streamlined process management and data analysis.

Other limitations of the study can be due to the online methodology (E-Delphi), which might seem slightly sterile lacking face-to-face debate and communication when dealing with animal welfare issues, creating potential misinterpretation of opinions [28]. On the other hand, this method allows participants to take time to answer and in-depth thinking while ensuring anonymity, information exchange, and opinion expression without bias linked to in-presence influence. In addition, this technique, when compared to similar methods (group discussion, meetings, etc.), allows researchers to focus on a specific topic involving a larger number of experts [28]. These aspects enabled us to overcome potential bias within this study to investigate relevant welfare aspects to be addressed in zoos and aquaria for different taxa, by involving experts from different facilities, backgrounds, and fields.

The recruitment process relied on the snowball sampling technique, however this methodology might potentially introduce a further bias as participants with strong interests were more engaged in the process. Moreover, the initial imbalance of the number of experts representing all taxa proved difficulties in obtaining taxa-specific responses. Participant fatigue is recognized as a Delphi limitation [46] and it has emerged in this study as a piece of evidence towards the end, despite an effort to minimize participant disengagement.

Despite the challenges and limitations linked to the Delphi method and its application underlined here, this study allowed us to investigate for the first time experts’ opinions about zoo animal welfare and reach a consensus in building a first list of questions to assess animal welfare in Italian zoos. Our questionnaire represents a starting point for Italian zoos to include animal welfare in their self-evaluation process and for competent authority in the assessment work, thus facilitating both. Future developments should try to address specific issues for each taxa, ensuring more details in assessing animal welfare and meeting taxa-specific needs. Further effort is required by the scientific community to fill the knowledge gap concerning the welfare needs of specific taxa and species. Future developments should provide a continuous and constant update of the questions and future processes to establish and test official checklists (every five years) to include new knowledge and scientific information while it is provided. In addition, since zoos play an active role in conservation, particular attention should be given to endangered species needs, focusing on conservation status, reintroduction programs, breeding programs, and related biosecurity as foreseen in a One Welfare–One Health approach. This would guarantee more detailed attention to ensure the health and welfare of the individuals, their reproduction, and the persistence of the species over time.

## Conclusion

This study was designed to explore the possibility of creating a practical toolkit for animal welfare provision and monitoring in Italian zoos and aquariums. The E-Delphi technique has proven to be a valuable methodology for collecting multi-stakeholder group opinions even on such a complex topic as the welfare of animals kept in zoos. This Delphi technique enabled us to obtain three distinct questionnaires categorized by items that, once validated through testing, could then be utilized to create a practical toolkit to assess adequate housing conditions for animals, while ensuring beneficial interaction between animals, trained staff, and visitors. Despite the E-Delphi technique requiring a very strong effort in terms of preparation and communication of the different steps to reach a significant consensus, it demonstrated to be a valuable methodology for collecting multi-stakeholder group opinions on animals’ welfare conditions in Italian zoos and aquaria. Overall, the Delphi study provided beneficial insights for practitioners in animal welfare in Italy: the resulting lists of questions to be tested and then implemented in Italian zoos could then provide contributions for other institutions located in other countries as well as academia, monitoring authorities, and policymakers. In conclusion, this study represents an effort in our commitment to ensure zoological gardens and aquariums’ animals a life worth living, with the permanent mission to restore their wild population with healthy, reactive, and adapted individuals.

The relevance of this study is further supported by the participation of the Italian National Reference Center for Animal Welfare, Istituto Zooprofilattico Sperimentale Institute of Lombardy and Emilia Romagna (IZSLER Brescia) which contributed to funding the study through the Italian Ministry of Health (IZSLER 02/18 RC Realization of a toolkit to evaluate animal welfare in zoos).

## Supporting information

S1 Table Definitions. (PDF)

S2 Table Topics that are not specifically included in the current Italian law. (DOCX)

S3 Table List of questions for cluster care. (DOCX)

S4 Table List of questions for cluster Wellbeing. (DOCX)

S5 Table List of questions for cluster Regulation. (DOCX)

S1 Dataset Round One results: Indicators. (XLSX)

S2 Dataset Round One results: Taxonomic groups’ relevance. (XLSX)

S3 Dataset Round Two results. (XLSX)

S4 Dataset Round Three results. (XLSX)
