# Supplementary material for: An E-Delphi study to facilitate animal welfare assessment in Italian zoos and aquaria
Source: PLoS One. 2025 Jan 6;20(1):e0309760. doi: 10.1371/journal.pone.0309760 (PMC11703047; doi:10.1371/journal.pone.0309760)
Supplement: S1 Table — (PDF) [file pone.0309760.s001.pdf]

Table of definitions of terms used through the Delphi process and in the list of questions

*The original language used throughout the study and in the list of questions is Italian, thus some slightly meaning differences can derive from translation in English.

Term	Definition
Theme	Macrocategory that propose a subject on which base the fundamental development lines of a wider discourse
Topic	Inside each theme, a topic is a more specific subcategory focused on a particular subject of that theme
Protocol	The official standardized set of rules and plan to implement a procedure, a process, and deal with different situations, formerly written as a specific document.
Procedure	A set of actions or methods used to accomplish a particular task, the official or accepted way of doing something.
Interactions with visitors	Experiences offered by zoos, sanctuaries, and other tourism facilities in which people can be very close, and even touch, wildlife (de Mori et al., 2019; Pollastri et al., 2022). (These activities can promote conservational and educational content, raise conservation mindedness and responsibility for the environment and animal welfare, but if not properly managed can jeopardize visitors' and animals' well-being and conservation efforts.)
General oganic functions	Functions that guarantee organism survival through the functionality of the different organs: from oxygen apport, to its distribution, metabolism, gastrointestinal and muscular function, etc.
Individual record	It contains the identification of the animal, and the history of clinical and ethological (behaviuoral observations) records.
Medical record	It contains the medical history, clinical examination, diagnostic investigations, therapy given and related to an individual. It is compiled by the veterinary, and may consider in medical history the history of individual clinical events reported previously.
Training	Contemporary animal training is a procedure to teach animals specific responses associated to specific conditioning or stimuli with the use of different types of reinforcement. Within modern zoo training is being associated with behavioral welfare advances such as: to increase voluntary husbandry care, implement environmental enrichment by promoting natural behaviours and also to facilitate animal acclimation to new environmental and social conditions [1]<br>The EAZA Animal Training Guidelines [2] define training as "intentionally changing behaviour with an awareness and understanding of the principles of behaviour analysis and applying these principles with individuals or groups of animals in managed care."

1. Fernandez EJ, Martin AL. Animal Training, Environmental Enrichment, and Animal Welfare: A History of Behavior Analysis in Zoos. Journal of Zoological and Botanical Gardens. 2021 Dec;2(4):531–43.
2. Heidenreich B, Pedersen A, Mackie J, Harding L. EAZA Animal Training Guidelines – 1st Edition [Internet]. 2023. Available from: https://www.eaza.net/assets/Uploads/CCC/BPG-2023/EAZA-ATWG-Training-Guidelines-3.pdf

1.
