# Supplementary material for: An E-Delphi study to facilitate animal welfare assessment in Italian zoos and aquaria
Source: PLoS One. 2025 Jan 6;20(1):e0309760. doi: 10.1371/journal.pone.0309760 (PMC11703047; doi:10.1371/journal.pone.0309760)
Supplement: S2 Table — (DOCX) [file pone.0309760.s002.docx]

| **Topics that are not explicitly cited in the current Italian Legislative decree n. 73 [15]** | |
| --- | --- |
| Cr7 | Substrate or flooring suited to the needs and characteristics of the hosted species |
| Cr11 | Freedom to access to indoor enclosures providing shelter from adverse weather conditions |
| Cr13 | Complexity of the enclosure environment allowing animals to engage in species-specific behaviours |
| Cr36 | Appropriate choice of species and individuals to be included in the mixed exhibits |
| Cr40 | Management of new entries |
| Cr63 | Management of individual animal welfare in breeding programs |
| Cr72 | Regular monitoring and evaluation of animal training |
| Cr73 | Source of the information used to make decisions regarding the management of animals for the aspects of the "care" area |
| Nv35 | Plan for the management of infectious and parasitic diseases, including corrective and preventive actions |
| Nv36 | Zoonosis risk assessment |
| Nv37 | Corrective and preventive actions taken in case of zoonosis |
| Nv40 | Cleaning and disinfection of the premises used for necropsy examinations |
| Nv42 | Groups of animals identification |
| Nv58 | Training and updating of staff on internal procedures |
| Nv59 | Training and updating of staff on animal health, welfare, and their monitoring |
| Nv60 | Specific training for staff handling animals in quarantine/isolation |
| Nv66 | Continuous professional development of keepers |
| Nv68 | Specific training on behavioural monitoring and species ethological repertoire for the cared species |
| Nv69 | Training on the design and use of enrichments |
| Nv70 | Training in monitoring environmental parameters in exhibits and tanks |
| Nv71 | Specific training for staff responsible for training, animal-visitor interaction and/or exhibitions |
| Wb1 | Preliminary evaluation before introducing a new species to the facility |
| Wb2 | Preliminary evaluation before acquiring new specimen |
| Wb11 | Enrichment monitoring |
| Wb4 | Inclusion of new individuals in compatible social groups |
| Wb37 | Prohibition of separating offspring from the mother solely for the purpose of learning for exhibitions and/or interactions with visitors |
| Wb40 | Prohibition of inducing animals to behave in a way that is contrary to the educational purposes of the interaction and/or performances |
