# Supplementary material for: An E-Delphi study to facilitate animal welfare assessment in Italian zoos and aquaria
Source: PLoS One. 2025 Jan 6;20(1):e0309760. doi: 10.1371/journal.pone.0309760 (PMC11703047; doi:10.1371/journal.pone.0309760)
Supplement: S3 Table — (DOCX) [file pone.0309760.s003.docx]

**LIST OF QUESTIONS - CARE**

| **N.** | **Question code (theme.indicator)** | **Question** |
| --- | --- | --- |
| **1** | **Cr1.1** | Are suitable facilities available to host injured or isolated animals based on the needs of different species? |
| **2** | **Cr1.2** | Is there space for staff to operate on animals within the facilities housing injured or isolated animals? |
| **3** | **Cr2.1** | Is there a procedure for managing pregnant females and offspring following EAZA guidelines and/or equivalent scientific literature and/or guidance from the responsible curator/veterinarian? |
| **4** | **Cr2.2** | Does the protocol for managing pregnant females and offspring include controlling access to isolation facilities? |
| **5** | **Cr3.1** | Are the enclosures and tanks hosting the animals of adequate size to allow for normal physiological behaviours following EAZA guidelines and/or equivalent scientific literature and/or guidance from the responsible curator/veterinarian? |
| **6** | **Cr4.1** | Does the animal density in the enclosures and/or tanks adhere to EAZA guidelines and/or equivalent scientific literature and/or guidance from the responsible curator/veterinarian? |
| **7** | **Cr4.1** | In the absence of species-specific guidelines, is the adequacy of animal density in the enclosures and/or tanks assessed by the curator and/or veterinary staff? |
| **8** | **Cr4.2** | Are shelters/refuges provided in the enclosures and/or tanks? |
| **9** | **Cr6.1**  **Cr39.2** | Are there separated areas within the enclosures and/or tanks in case of necessity? |
| **10** | **Cr6.1** | Are there protocols in place for separating animals in case of necessity? |
| **11** | **Cr6.2** | Are there areas where animals can be housed without public access? |
| **12** | **Cr7.1** | Is the flooring or substrate of the environments designed to meet the needs of the species following EAZA guidelines and/or equivalent scientific literature and/or guidance from the responsible curator/veterinarian? |
| **13** | **Cr7.2** | Is there a protocol for the removal of excreta, partial and total substrate replacement, along with disinfection of litter/floors? |
| **14** | **Cr8.1** | Does the substrate allow for water drainage? |
| **15** | **Cr8.2** | Do the flooring and surfaces allow for water drainage with slopes and drains? |
| **16** | **Cr9.1** | Is the material used for furnishings in the enclosures suitable for the physiological and ethological characteristics of the species following EAZA guidelines and/or equivalent scientific literature and/or guidance from the responsible curator/veterinarian? |
| **17** | **Cr10.1** | Are there an adequate number of shelters for adverse weather conditions in the outdoor enclosures following EAZA guidelines and/or equivalent scientific literature and/or guidance from the responsible curator/veterinarian? |
| **18** | **Cr10.2** | Are there heated shelters for adverse weather conditions in the outdoor enclosures following EAZA guidelines and/or equivalent scientific literature and/or guidance from the responsible curator/veterinarian? |
| **19** | **Cr10.2** | Is there a protocol outlining the procedures for accessing the heated shelters? |
| **20** | **Cr10.3** | For species with specific temperature and humidity requirements, are these needs met by monitoring them with specific equipment? |
| **21** | **Cr10.3**  **Cr23.2** | Are there adequate tools for monitoring and recording temperature and humidity in the enclosures and/or tanks? |
| **22** | **Cr11.1** | Is the indoor enclosure easily inspectable by staff through independent access, with dimensions and characteristics following EAZA guidelines and/or equivalent scientific literature and/or guidance from the responsible curator/veterinarian? |
| **23** | **Cr11.1** | Is there the possibility of unrestricted access for staff to the indoor enclosures? |
| **24** | **Cr13.1** | Is the enclosure structured following EAZA guidelines and/or equivalent scientific literature and/or guidance from the responsible curator/veterinarian? |
| **25** | **Cr17.1** | Are there dens, shelters, and/or visual barriers to allow animals to avoid interactions with conspecifics? |
| **26** | **Cr17.1** | Are the enclosures arranged to allow animals to avoid contact with conspecifics? |
| **27** | **Cr18.1**  **Cr45.2** | Are there specific feeding programs for the housed species? |
| **28** | **Cr18.1**  **Cr36.2** | Is access to food and water ensured for all housed specimens? |
| **29** | **Cr20.1** | As for safety, are the enclosures structured following EAZA guidelines and/or equivalent scientific literature and/or guidance from the responsible curator/veterinarian regarding safety considerations? |
| **30** | **Cr20.1** | Do the tanks and enclosures ensure the safety of the animals in terms of technical specifications and hygiene-sanitary conditions? |
| **31** | **Cr20.2** | Do the enclosures have surfaces that allow for safe cleaning and disinfection operations? |
| **32** | **Cr20.3** | Is access to the enclosures regulated by the presence of deterrent signs, locks, padlocks, and barriers? |
| **33** | **Cr20.4** | Is the enclosure secure, considering the characteristics of the hosted species? |
| **34** | **Cr20.5** | Are there containment means for the hosted species, and are they in good working condition? |
| **35** | **Cr20.6** | Is there an emergency plan to manage the potential escapes of animals based on the species involved? |
| **36** | **Cr22.1** | Is there an access protocol for personnel responsible for managing areas where dangerous species are housed? |
| **37** | **Cr22.1** | Are measures adopted to ensure the safety of personnel involved in the management of dangerous species? |
| **38** | **Cr23.1** | Are the temperature and humidity levels in the enclosures following EAZA guidelines and/or equivalent scientific literature and/or guidance from the responsible curator/veterinarian? |
| **39** | **Cr25.1** | Does the ventilation system follow EAZA guidelines and/or equivalent scientific literature and/or guidance from the responsible curator/veterinarian? |
| **40** | **Cr26.1** | Are the chemical and physical parameters of the water in the tanks regularly monitored? |
| **41** | **Cr26.1** | Are the chemical and physical parameters of the water in the tanks under EAZA guidelines and/or equivalent scientific literature and/or guidance from the responsible curator/veterinarian? |
| **42** | **Cr26.2** | Is there a report that records the chemical, physical, and microbiological parameters of the water in the tanks? |
| **43** | **Cr26.2** | Are periodic evaluations conducted on the chemical, physical, and microbiological parameters of the water hosting fish or marine mammals? |
| **44** | **Cr27.1** | Does the type of lighting follow the EAZA guidelines and/or equivalent scientific literature and/or guidance from the responsible curator/veterinarian? |
| **45** | **Cr27.1** | Does the lighting correspond to the physio-ethological needs of the species? |
| **46** | **Cr27.2** | Are backup systems in place in case of a lighting system malfunction? |
| **47** | **Cr28.1** | Is adequate lighting provided during the winter months based on species-specific needs? |
| **48** | **Cr36.1** | Is the establishment of mixed exhibits conceived following EAZA guidelines and/or equivalent scientific literature and/or guidance from the responsible curator/veterinarian? |
| **49** | **Cr36.1** | Are mixed exhibits designed taking into consideration the physio-ethological characteristics of the species? |
| **50** | **Cr37.1** | Are there species-specific accessible zones to allow animals to avoid interaction with individuals of other species? |
| **51** | **Cr40.1** | Are new introductions carried out following EAZA guidelines and/or equivalent scientific literature and/or guidance from the responsible curator/veterinarian? |
| **52** | **Cr51.1** | Is there an evaluation by the curator and veterinary staff of any targeted sterilization programs? |
| **53** | **Cr51.1** | Is there a plan for birth control? |
| **54** | **Cr51.2** | Is there an environmental enrichment plan for the species housed in the mixed exhibits? |
| **55** | **Cr43.1**  **Cr43.2** | Are periodic inspections carried out on trees inside or near the enclosures to reduce the risk of injuries and/or escapes of the animals? |
| **56** | **Cr44.1** | Do the methods of food and water administration take into account the species-specific ethological characteristics following EAZA guidelines and/or equivalent scientific literature and/or guidance from the responsible curator/veterinarian? |
| **57** | **Cr44.1** | Are there food administration protocols that take into account species-specific characteristics? |
| **58** | **Cr44.2**  **Cr44.4** | Is the staff trained on the ethological characteristics of the species they are caring for? |
| **59** | **Cr44.3** | Is there a rodent control and biological vector management program in place? |
| **60** | **Cr44.4** | Is there a feeding plan for the housed animals that takes into account seasonal variations in their diet? |
| **61** | **Cr45.1** | If necessary, are there specific diets for individual animals following EAZA guidelines and/or equivalent scientific literature and/or guidance from the responsible curator/veterinarian? |
| **62** | **Cr45.2** | Are there species-specific feeding plans in place? |
| **63** | **Cr45.3** | Are diets prepared following the existing feeding plans? |
| **64** | **Cr50.1** | Is there a procedure for managing the potential relocation of individuals to other facilities in case of conflict between individuals of the same species? |
| **65** | **Cr50.2** | Is training also used to reduce conflicts between individuals of the same species? |
| **66** | **Cr53.1** | Is there a veterinary protocol for managing pregnant or lactating females? |
| **67** | **Cr50.2** | Is the staff adequately trained in handling pregnant or lactating females? |
| **68** | **Cr54.1** | Is there a preventive medicine plan for pregnant females and newly born animals? |
| **69** | **Cr54.1** | Is there a schedule for veterinary visits for pregnant females and newly born animals? |
| **70** | **Cr73.1** | Does the staff base their decisions regarding animal management on scientific literature? |
| **71** | **Cr73.2** | Do the technical staff and veterinarians make decisions regarding animal management by updating themselves and consulting with experts in the field at the national and/or international level? |
| **72** | **Cr73.3** | Is there periodic training for the staff on international standards and regulatory updates? |
| **73** | **Cr73.4** | Are there regular internal meetings for updates and planning on animal management? |
| **74** | **Cr64.1** | In birth planning, are the recommendations of the EEP coordinators for species included in such programs followed to ensure the long-term welfare of the animals? |
| **75** | **Cr64.2** | Are there reproduction and contraception programs for species not included in EEP programs that ensure the long-term welfare of the animals? |
| **76** | **Cr64.3** | Does the facility participate in species conservation programs at local, national, and/or international levels? |
| **77** | **Cr65.1** | Is there a training protocol specifying the type and method for conducting different training sessions? |
| **78** | **Cr65.1** | If there is a training protocol in place, does it include monitoring and control of progress? |
| **79** | **Cr65.2** | If there is a training protocol in place, is it structured with annual/semi-annual scheduling? |
| **80** | **Cr65.2** | If there is a training protocol in place, is its implementation periodically verified? |
| **81** | **Cr65.3** | Is the staff trained to carry out the activities outlined in the training program? |
| **82** | **Cr65.3** | Is the staff regularly updated to implement the activities outlined in the training program? |
| **83** | **Cr72.1** | Is there a procedure to evaluate the progress and level of participation of the animals during training? |
| **84** | **Cr72.1** | Does the training protocol include monitoring of sessions through observations? |

**Themes and indicators’ codes:**

Cr 1 - Theme SUITABLE FACILITIES OR STRUCTURES FOR THE CARE OF INJURED, ILL, OR STRESSED ANIMALS

**Indicators**:

1. ***Presence of hospital rooms and isolation rooms***
2. ***Adequate surface for inspection and handling of animals by technical and medical staff***

Cr2 - Theme SUITABLE ENCLOSURES AND TANKS FOR PREGNANT FEMALES OR THOSE RAISING OFFSPRING

**Indicators:**

1. ***Dimensions of species-specific rooms as indicated by EAZA guidelines where available or by other scientific literature. In the absence of guidelines, evaluation of dimensions based on legal standards and decisions by curator and veterinary staff.***
2. ***Restricted access to hospital and isolation rooms to technical and veterinary staff.***

Cr3 - Theme APPROPRIATE ENCLOSURES OR TANKS DIMENSIONS ACCORDING TO THE PHYSICAL AND BEHAVIOURAL CHARACTERISTICS OF THE HOUSED ANIMALS

**Indicators:**

1. ***Species-specific enclosure dimensions as indicated by EAZA guidelines where available or by other scientific literature. In the absence of guidelines, decisions by curator and veterinary staff.***

Cr4 - Theme APPROPRIATE ANIMAL DENSITY IN ENCLOSURES AND/OR TANKS ACCORDING TO THE PHYSICAL AND BEHAVIOURAL CHARACTERISTICS OF THE HOUSED ANIMALS.

**Indicators:**

1. ***Species-specific enclosure dimensions as indicated by EAZA guidelines where available or by other scientific literature. In the absence of guidelines, decisions by curator and veterinary staff.***
2. ***Presence of sheltered areas.***

Cr6 - Theme POSSIBILITY TO SEPARATE INDIVIDUAL ANIMALS OR SMALL GROUPS OF ANIMALS IN CASE OF NECESSITY

**Indicators:**

1. ***The presence of suitable dividers, fences, connections, and tunnels resulting in the modularity of the structure.***
2. ***Availability of enclosures for animals not visible to the public.***

Cr7 - Theme SUBSTRATE OR FLOORING SUITED TO THE NEEDS AND CHARACTERISTICS OF THE HOSTED SPECIES

**Indicators:**

1. ***Evaluation and selection of substrate as indicated by EAZA guidelines where available or by other scientific literature. In the absence of guidelines, the curator and veterinary staff evaluate based on legal standards and decisions.***
2. ***A clear description of substrate management procedures (cleaning, replacement, and supplementation) by staff.***

Cr8 - Theme SURFACES AND FLOORING THAT ALLOW FOR PROPER WATER DRAINAGE

**Indicators:**

1. ***Evaluation and selection of substrate as indicated by EAZA guidelines where available or by other scientific literature.***
2. ***Presence of slopes and drains in indoor enclosures.***

Cr9 - Theme APPROPRIATE PROVISION OF MATERIALS AND STRUCTURES (BEDDING, DENS, NEST BOXES, ETC.) THAT ALLOW FOR THE EXPRESSION OF SPECIES-SPECIFIC BEHAVIOURS.

**Indicators:**

1. ***Planning according to EAZA guidelines where available or other scientific literature. In the absence of guidelines, the curator and veterinary staff evaluate based on legal standards and decisions.***

Cr10 - Theme ADEQUACY OF SHELTERS FOR ADVERSE WEATHER CONDITIONS IN OUTDOOR ENCLOSURES

**Indicators:**

1. ***Planning according to EAZA guidelines where available or other scientific literature. In the absence of guidelines, the curator and veterinary staff evaluate based on legal standards and decisions.***
2. ***Presence of heating accessory where necessary.***
3. ***Adequate maintenance of temperature and humidity inside shelters and possible monitoring of them through suitable equipment.***

Cr11 - Theme FREEDOM TO ACCESS TO INDOOR ENCLOSURES PROVIDING SHELTER FROM ADVERSE WEATHER CONDITIONS.

**Indicators**:

1. ***Presence of an indoor enclosure easily inspectable by staff through independent access. In the absence of guidelines, the curator and veterinary staff evaluate based on legal standards and decisions.***

Cr13 - Theme COMPLEXITY OF THE ENCLOSURE ENVIRONMENT ALLOWING ANIMALS TO ENGAGE IN SPECIES-SPECIFIC BEHAVIOURS

**Indicators:**

1. ***Planning according to EAZA guidelines where available or other scientific literature. In the absence of specific guidelines, evaluations by the curator and veterinary staff.***

Cr17 - Theme OPPORTUNITIES FOR ANIMALS TO AVOID INTERACTIONS WITH CONSPECIFICS

**Indicators:**

1. ***Presence of dens, shelters, or visual barriers - Planning according to EAZA guidelines where available or other scientific literature. In the absence of specific guidelines, evaluations by the curator and veterinary staff - Evaluation of the complexity and suitability of the exhibit concerning the needs of the housed species***

Cr18 - Theme GUARANTEED ACCESS FOR ALL ANIMALS SIMULTANEOUSLY TO FOOD AND WATER RATIONS

**Indicators:**

1. ***Feeding of animals according to the dietary habits of the species.***

Cr20 - Theme APPROPRIATE SAFETY OF ENCLOSURES AND TANKS REGARDING HOUSED ANIMALS

**Indicators:**

1. ***Utilization of EAZA guidelines where available or other scientific literature with evaluation of any species-specific parameters. In the absence of guidelines, the curator and veterinary staff evaluate based on legal standards and decisions.***
2. ***Ensure easy cleaning and disinfection and durability of materials.***
3. ***Presence of locks, padlocks, and gates to reduce accessibility.***
4. ***Verification of the facility's characteristics regarding the potential danger of the individual housed species.***
5. ***Periodic inspection of the good condition of animal containment devices.***
6. ***Application of management protocol to prevent possible escapes.***

Cr22 - Theme SUITABILITY OF FENCES AND BARRIERS FOR CONTAINING ANIMALS THAT POSE A RISK TO PUBLIC HEALTH AND SAFETY

**Indicators:**

1. ***Ensure staff safety as required by law.***

Cr23 - Theme TEMPERATURE AND HUMIDITY LEVELS OF ENCLOSURES AND TANKS SUITABLE FOR THE COMFORT AND WELL-BEING OF THE HOUSED ANIMALS

**Indicators:**

1. ***Utilization of EAZA guidelines where available or other scientific literature with evaluation of any species-specific parameters. In the absence of guidelines, the curator and veterinary staff evaluate based on legal standards and decisions.***
2. ***Temperature control - Humidity monitoring and control for relevant species.***

Cr25 - Theme VENTILATION OF THE ENCLOSURE SUITABLE FOR THE COMFORT AND WELL-BEING OF THE HOUSED ANIMALS

**Indicators:**

1. ***Utilization of EAZA guidelines where available or other scientific literature with evaluation of any species-specific parameters. In the absence of guidelines, the curator and veterinary staff evaluate based on legal standards and decisions*.**

Cr26 - Theme APPROPRIATE CHEMICAL-PHYSICAL PARAMETERS OF WATER IN THE TANKS BASED ON THE NEEDS OF THE HOSTED SPECIES

**Indicators:**

1. ***Utilization of EAZA guidelines where available or other scientific literature with evaluation of any species-specific parameters. In the absence of guidelines, the curator and veterinary staff evaluate based on legal standards and decisions*.**
2. ***Evaluation of chemical, physical, and microbiological parameters of water to be carried out regularly.***

Cr27 - Theme QUANTITY AND TYPE OF LIGHTING SUITABLE FOR THE COMFORT AND WELL-BEING OF THE HOUSED ANIMALS

**Indicators:**

1. ***Utilization of EAZA guidelines where available or other scientific literature with evaluation of any species-specific parameters. In the absence of guidelines, the curator and veterinary staff evaluate based on legal standards and decisions*.**
2. ***Presence of spare lamps to be used in case of a lamp breakage.***

Cr28 - Theme APPROPRIATE ALTERNATION OF LIGHT AND DARK PERIODS THAT RESPECT THE NEEDS OF THE HOUSED ANIMALS

**Indicators:**

1. ***Presence of adequate artificial lighting during the winter months for animals not accustomed to long periods of darkness.***

Cr36 - Theme APPROPRIATE SELECTION OF SPECIES AND INDIVIDUALS TO BE INCLUDED IN MIXED EXHIBITS

**Indicators:**

1. ***Utilization of EAZA guidelines where available or other scientific literature with evaluation of any species-specific parameters. In the absence of guidelines, the curator and veterinary staff evaluate based on legal standards and decisions*.**
2. ***Access to water and food for all individuals.***

Cr37 - Theme OPPORTUNITIES FOR ANIMALS TO AVOID INTERACTION WITH INDIVIDUALS OF OTHER SPECIES PRESENT

**Indicators:**

1. ***Presence of reserved areas accessible in a species-specific manner.***

Cr39 - Theme MANAGEMENT OF POTENTIAL HIERARCHIES

**Indicators:**

1. ***Utilization of EAZA guidelines where available or other scientific literature with evaluation of any species-specific parameters. In the absence of guidelines, the curator and veterinary staff evaluate based on legal standards and decisions*.**
2. ***Possibility to create subgroups or isolate weaker animals.***

Cr40 - Theme MANAGEMENT OF NEW INTRODUCTIONS

**Indicators:**

1. ***Utilization of EAZA guidelines where available or other scientific literature with evaluation of any species-specific parameters. In the absence of guidelines, the curator and veterinary staff evaluate based on legal standards and decisions*.**

Cr51 - Theme MANAGEMENT OF CONFLICTS AMONG INDIVIDUALS WITHIN MIXED EXHIBITS

**Indicators:**

1. ***Implementation of scheduled sterilizations.***
2. ***Presence of environmental enrichments.***

Cr43 - Theme REGULAR INSPECTIONS OF TREES INSIDE THE ENCLOSURES OR THOSE NEAR ENCLOSURES TO REDUCE THE RISK OF INJURIES AND/OR ESCAPES OF ANIMALS.

**Indicators:**

1. ***Periodic checks of trees and analysis of their condition and size.***
2. ***Inspections conducted by keepers.***

Cr44 - Theme FEEDING AND WATERING METHODS THAT TAKE INTO UTMOST CONSIDERATION THE BEHAVIOUR OF THE ANIMALS

**Indicators:**

1. ***Utilization of EAZA guidelines where available or other scientific literature with evaluation of any species-specific parameters. In the absence of guidelines, the curator and veterinary staff evaluate based on legal standards and decisions*.**
2. ***Species ethology knowledge.***
3. ***Presence of a pest management and monitoring program.***
4. ***Diet variation according to seasonality.***

Cr45 - Theme BALANCED DIET QUANTITATIVELY AND QUALITATIVELY RESPECTING THE NEEDS OF THE INDIVIDUAL

**Indicators:**

1. ***Utilization of EAZA guidelines where available or other scientific literature with evaluation of any species-specific parameters. In the absence of guidelines, evaluation based on legal standards and decisions by the curator and veterinary staff*.**
2. ***Presence of a feeding protocol with diets dedicated to each species and specific types of supplements used.***
3. ***Adherence to diets formulated by a veterinarian/curator.***

Cr50 - Theme MANAGEMENT OF CONFLICTS AMONG INDIVIDUALS OF THE SAME SPECIES

**Indicators:**

1. ***Plans for transferring animals to other facilities if compatibility issues among individuals are identified.***
2. ***Trainings of the individuals***

Cr53 - Theme ACQUISITION AND IMPLEMENTATION OF PRESCRIPTIONS FROM THE VETERINARIAN AND/OR THE CURATOR OF THE AQUARIUM FOR THE ACCOMMODATION OR TRANSFER OF PREGNANT OR LACTATING FEMALES.

**Indicators:**

1. ***Utilization of veterinary guidelines where available, or other scientific literature, with assessment of any species-specific parameters.***
2. ***Presence of staff capable of adhering to the veterinarian's/curator's instructions.***

Cr54 - Theme MANAGEMENT OF PREGNANT FEMALES, NEAR BIRTH, AND NEWLY BORN ANIMALS.

**Indicators:**

1. ***Constant monitoring of the health status of the animals with veterinary check-ups***

Cr73 - Theme SOURCE OF THE INFORMATION USED TO MAKE DECISIONS REGARDING THE MANAGEMENT OF ANIMALS FOR THE ASPECTS OF THE "CARE" AREA

**Indicators:**

1. ***Extraction of information from peer-reviewed journals and publications in the field.***
2. ***Updates and international comparisons by technical and veterinary medical staff.***
3. ***Knowledge of international standards and presence of an internal repository for constant regulatory updates.***
4. ***Holding periodic internal meetings.***

Cr64 - Theme DEVELOPMENT OF BREEDING PROGRAMS ENSURING THE LONG-TERM WELFARE OF THE ANIMALS.

**Indicators:**

1. ***Following EEP coordinator recommendations, where applicable***
2. ***Avoid cases of inbreeding***
3. ***Adherence to local, national, and international conservation and breeding programs.***

Cr65 - Theme ADEQUATE ANIMAL TRAINING (Frequency and duration of sessions, type of positive reinforcement used)

**Indicators:**

1. ***Presence of a training protocol with adequate recording of sessions***
2. ***Verification of annual/semester training program***
3. ***Staff training***

Cr72 - Theme REGULAR MONITORING AND EVALUATION OF ANIMAL TRAINING

**Indicators:**

1. ***Monitoring the level of animal participation in training sessions***
