# Supplementary material for: An E-Delphi study to facilitate animal welfare assessment in Italian zoos and aquaria
Source: PLoS One. 2025 Jan 6;20(1):e0309760. doi: 10.1371/journal.pone.0309760 (PMC11703047; doi:10.1371/journal.pone.0309760)
Supplement: S4 Table — (DOCX) [file pone.0309760.s004.docx]

**LIST OF QUESTIONS - WELLBEING**

| **N.** | **Question code (theme.indicator)** | **Question** |
| --- | --- | --- |
| **1** | **Wb1.1** | Is there a protocol regarding the procedures for introducing a new species into the facility? |
| **2** | **Wb1.2** | Before acquiring a new species, a is a preliminary evaluation conducted following the EAZA guidelines and/or equivalent standards and/or scientific literature and/or recommendations from the curator/veterinarian in charge. |
| **3** | **Wb2.1** | Is the periodic census of animals carried out following the law? |
| **4** | **Wb2.1** | Is there a real-time updated database containing the list of all animals? |
| **5** | **Wb2.2** | Is there a sanitary isolation/quarantine area following EAZA guidelines and/or equivalent standards and/or scientific literature and/or indications from the curator/responsible veterinarian? |
| **6** | **Wb2.3** | Is there an environmental enrichment program following EAZA guidelines and/or equivalent standards and/or scientific literature and/or indications from the curator/responsible veterinarian? |
| **7** | **Wb2.4** | Is there a protocol for evaluating the compatibility of new individuals with the existing group of animals following EAZA guidelines and/or equivalent standards and/or scientific literature and/or indications from the curator/responsible veterinarian? |
| **8** | **Wb2.4** | Before arrival at the facility, has there been an assessment of the compatibility of the new animals with the existing group of animals following EAZA guidelines and/or equivalent standards and/or scientific literature and/or indications from the curator/responsible veterinarian? |
| **9** | **Wb2.5** | Before the introduction of new individuals, in addition to following EAZA guidelines or similar, are the opinions of experts consulted (e.g., other facilities housing the species)? |
| **10** | **Wb4.1** | Are there confined spaces of suitable dimensions to facilitate the introduction of new specimens following EAZA guidelines or equivalent, scientific literature, or indications from the curator/veterinarian in charge? |
| **11** | **Wb11.1** | Are enrichments monitored using methods following the EAZA guidelines or equivalent, scientific literature, or indications from the curator/veterinarian in charge? |
| **12** | **Wb29.1** | Are there monitoring sheets for visitor activity? |
| **13** | **Wb29.1** | During the interaction, are there shelters/refuges that provide animals with the opportunity to move away from visitors? |
| **14** | **Wb29.2** | Is there a protocol describing the behaviour to be observed by visitors participating in interactions, following EAZA guidelines or equivalent, scientific literature, or indications from the responsible curator/veterinarian? |
| **15** | **Wb29.2** | Is there a methodology in place to monitor compliance with the protocol? |
| **16** | **Wb29.2** | Are there signs and indications informing visitors about the rules of conduct? |
| **17** | **Wb37.1** | Are the guidelines of EAZA or equivalent, scientific literature, or indications from the curator/veterinarian responsible applied regarding the prohibition of separating offspring from the mother solely to learn for exhibitions and/or interactions with visitors? |
| **18** | **Wb40.1** | Are the guidelines of EAZA or equivalent, scientific literature, or indications from the curator/veterinarian responsible applied regarding the prohibition of inducing animals to exhibit behaviours that are contrary to the educational purposes of the interaction and/or exhibitions? |

**Themes and indicators’ codes:**

Wb 1 - Theme PRELIMINARY EVALUATION BEFORE INTRODUCING A NEW SPECIES TO THE FACILITY

***Indicators:***

1. ***Presence of a project outlining the methods for the new species introduction into the facility (exhibit design, trained personnel, safety measures, etc.)***
2. ***Application of EAZA guidelines or similar where available, and consultation of current literature. In the absence of specific guidelines, evaluation by the curator and veterinary staff.***

Wb 2 - Theme PRELIMINARY EVALUATION BEFORE INTRODUCING A NEW SPECIMEN TO THE FACILITY

***Indicators:***

1. ***Carrying out periodic censuses as required by law***
2. ***Presence of a quarantine area following EAZA guidelines or similar. In the absence of specific guidelines, evaluation by the curator and veterinary staff***
3. ***Presence of environmental enrichment as indicated by EAZA guidelines or similar. In the absence of specific guidelines, evaluation by the curator and veterinary staff***
4. ***Evaluation of compatibility with existing animal groups as indicated by EAZA guidelines or similar. In the absence of specific guidelines, evaluation by the curator and veterinary staff***
5. ***Consultation of expert opinions (e.g., other facilities housing the species)***

Wb 4 - Theme INTRODUCTION OF NEW SPECIMENS INTO COMPATIBLE SOCIAL GROUPS

***Indicators:***

1. ***Assessment of the presence of confined spaces to facilitate the introduction of new specimens as indicated by EAZA or similar guidelines. In the absence of specific guidelines, evaluation by the curator and veterinary staff.***

Wb 11 - Theme ENVIRONMENTAL ENRICHMENTS MONITORING

***Indicators:***

1. ***Presence of methods for recording the use of enrichments.***

Wb 29 - Theme OPPORTUNITY FOR ANIMALS TO INTERRUPT INTERACTIONS WITH VISITORS AND MOVE AWAY

***Indicators:***

1. ***Presence of a non-public accessible area, Shelter availability to shield from visitors' view***
2. ***Regulation of visitor behaviour guidelines.***

Wb 37 - Theme PROHIBITION OF SEPARATING OFFSPRING FROM THE MOTHER SOLELY FOR THE PURPOSE OF LEARNING FOR EXHIBITIONS AND/OR INTERACTIONS WITH VISITORS

***Indicators:***

1. ***Application of EAZA guidelines "Guidelines on the use of animals in public demonstrations sect 3: Health of animals". In the absence of specific guidelines, evaluation by the curator and veterinary staff.***

Wb 40 - Theme PROHIBITION OF INDUCING ANIMALS TO EXHIBIT BEHAVIOURS THAT ARE CONTRARY TO THE EDUCATIONAL PURPOSES OF THE INTERACTION AND/OR EXHIBITIONS

***Indicators:***

1. ***Application of EAZA guidelines "Guidelines on the use of animals in public demonstrations sect 3: Health of animals". In the absence of specific guidelines, evaluation by the curator and veterinary staff.***
