# Supplementary material for: An E-Delphi study to facilitate animal welfare assessment in Italian zoos and aquaria
Source: PLoS One. 2025 Jan 6;20(1):e0309760. doi: 10.1371/journal.pone.0309760 (PMC11703047; doi:10.1371/journal.pone.0309760)
Supplement: S5 Table — (DOCX) [file pone.0309760.s005.docx]

**LIST OF QUESTIONS - REGULATION**

| **N.** | **Question code (theme.indicator)** | **Question** |
| --- | --- | --- |
| **1** | **Nv2.1** | Is there a protocol for assessing the nutritional and hydration status of the animals? |
| **2** | **Nv2.1** | Is the assessment of the nutritional and hydration status of the animals conducted at predetermined intervals? |
| **3** | **Nv2.1** | Is there a protocol for managing the animals' dietary plan? |
| **4** | **Nv2.1** | Is the Body Condition Score (BCS) calculated for each hosted species where possible? |
| **5** | **Nv2.1** | Are the keepers trained to evaluate the Body Condition Score (BCS)? |
| **6** | **Nv2.2** | Is there a protocol for recording daily checks by the keepers? |
| **7** | **Nv2.2** | For the daily health check of the various species housed, are there daily recording plans/tables managed by the keepers? |
| **8** | **Nv2.2** | Do keepers regularly update the daily recording plans/tables? |
| **9** | **Nv2.2** | Is there a predetermined frequency for a veterinarian's site visit to verify the daily check recordings made by keepers? |
| **10** | **Nv2.3** | Is there a check of the overall health and behaviour of the various species housed? |
| **11** | **Nv2.4** | Are keepers instructed to check for the presence/absence/quantity of faeces based on the species? |
| **12** | **Nv2.4** | Are the major organ functions (respiration, feeding, urination, and defecation) observed daily? |
| **13** | **Nv2.5** | During the cleaning of the enclosure, food provision, and when environmental enrichments are provided, are anomalies in the reaction to external stimuli recorded? |
| **14** | **Nv2.5** | Are the keepers trained to recognize the normal reactivity of animals to daily stimuli? |
| **15** | **Nv2.5** | Do the animals react naturally to stimuli? |
| **16** | **Nv2.5** | Is the sensory state, the reactivity of the animal, and how it interacts with the surrounding space and other subjects evaluated? |
| **17** | **Nv2.6** | Are the keepers trained to monitor the health conditions of the animals based on their species? |
| **18** | **Nv2.6**  **Nv66.1** | Is there a training program and ongoing education for the keepers? |
| **19** | **Nv2.6** | Is the effectiveness of the daily animal management activities evaluated? |
| **20** | **Nv2.6** | Are there enough keepers relative to the number of individuals present? |
| **21** | **Nv2.7** | Is there a procedure for calling the veterinarian in case the keeper detects anomalies or changes in the health or behaviour of the animals? |
| **22** | **Nv2.7** | Is there a list of the staff members authorized to request the intervention of a veterinarian? |
| **23** | **Nv2.7** | Is there an archive of the veterinarian's interventions? |
| **24** | **Nv2.8**  **Nv7.2** | Are there records with reports, medical history, and individual medical records? |
| **25** | **Nv2.8** | Individual records containing medical history and clinical records are present and easily accessible |
| **26** | **Nv2.8** | Are the medical records updated by the veterinarian? |
| **27** | **Nv2.9** | When deemed possible by the veterinarian or keeper, are the animals' weights checked? |
| **28** | **Nv2.9** | During training, are the animals weighed? |
| **29** | **Nv2.9** | Is the result of weight checks recorded (on the individual record)? |
| **30** | **Nv2.10** | Is the intake of administered food within the enclosures/tanks monitored? |
| **31** | **Nv2.11** | Is there a protocol indicating which health condition checks of the animals should be recorded daily? |
| **32** | **Nv2.12** | Is there a procedure for monitoring the behaviour of the animals over time? |
| **33** | **Nv2.13** | Is the regular gastrointestinal function evaluated based on the species? |
| **34** | **Nv4.1** | Is there a registry of periodic diagnostic tests required by regulations and protocols? |
| **35** | **Nv4.1** | Is the registry of periodic diagnostic tests required by regulations and protocols easily accessible? |
| **36** | **Nv4.1** | Is there a program of routine diagnostic tests (not on call) carried out by veterinarians? |
| **37** | **Nv4.2** | Are necropsies performed on deceased animals? |
| **38** | **Nv4.2** | Are necropsies also accompanied by histological examinations? |
| **39** | **Nv4.2** | Is there a criterion (for all animals, randomly selected, based on suspicion, etc.) for deciding when to perform histological examinations? |
| **40** | **Nv4.2** | Do necropsies also include microbiological diagnostics? |
| **41** | **Nv4.3** | Is the health prophylaxis plan differentiated according to the different species? |
| **42** | **Nv4.3** | Does the health prophylaxis plan take into account the epidemiological situation of the territory? |
| **43** | **Nv7.1** | Are prophylactic and disease prevention interventions planned? |
| **44** | **Nv7.1** | Is there an annual plan for monitoring animal diseases that is available and accessible for consultation? |
| **45** | **Nv7.3** | Are there veterinarians available at the facility? |
| **46** | **Nv7.3** | Are there criteria for selecting veterinarians that take into account the type of training they have received? |
| **47** | **Nv7.3** | Is there a sufficient number of veterinarians at the facility? |
| **48** | **Nv7.3** | Is there an organizational chart of the employed veterinarians? |
| **49** | **Nv7.3** | Are the curricula of the veterinarians available for consultation? |
| **50** | **Nv8.1** | Is the veterinary staff clearly identified following current regulations according to Legislative Decree 73/2005? |
| **51** | **Nv8.2** | Is there veterinary assistance capable of ensuring timely intervention in case of emergency? |
| **52** | **Nv9.1** | Are there facilities and veterinary equipment for the clinical care of animals as indicated by current regulations? |
| **53** | **Nv10.1** | Does the individual clinical record contain complete information on each intervention, diagnosis, therapy, and re-evaluation of the subjects after reporting? |
|  | | |
| **54** | **Nv11.1**  **Nv11.4** | Is there an authorized stock of veterinary medicines? |
| **55** | **Nv11.1** | Is there a list of authorized veterinarians allowed to use the stock of veterinary medicines? |
| **56** | **Nv11.1** | Is the prompt availability of tranquillizers and anaesthetics ensured? |
| **57** | **Nv11.2** | Is there a protocol for managing the stock of veterinary drugs? |
| **58** | **Nv11.3** | Are the necessary equipment for administering narcotics available under safe conditions? |
| **59** | **Nv11.3** | Are there facilities for the safe awakening of the animal? |
| **60** | **Nv11.4** | Are the authorizations held by the facility issued by the competent ASL (Local Health Authority) for the possession and use of tranquillizers and anaesthetics available and accessible for consultation? |
| **61** | **Nv13.1** | Are there equipment and premises for the containment of animals if necessary? |
| **62** | **Nv14.1** | Is there a protocol for rapid communication by keepers of an impediment, a health or safety problem (to people or animals)? |
| **63** | **Nv14.2** | Does the facility invest in continuous training for its staff? |
| **64** | **Nv14.2**  **Nv18.3** | Is there documentation attesting to the staff's training at various levels, including details of refresher courses and years of experience? |
| **65** | **Nv64.1** | Is the safety of an animal ensured during containment, movement, and transportation operations through appropriate means? |
| **66** | **Nv64.1** | Is there a list of the main structures and equipment for the containment, capture, and transportation of animals? |
| **67** | **Nv64.1** | Is there a person responsible for animal handling and transportation operations? |
| **68** | **Nv64.1** | Are the transport vehicles regularly authorized under Regulation 1/2005? |
| **69** | **Nv64.1** | Is there a workplace safety plan in place? |
| **70** | **Nv18.1** | Is there dedicated staff at the facility for cleaning the enclosures? |
| **71** | **Nv18.1** | Is the cleaning of the enclosures carried out at regular and predetermined intervals? |
| **72** | **Nv18.1** | Are the cleaning activities of the enclosures recorded? |
| **73** | **Nv18.1** | Is there a protocol for evaluating the cleanliness of the enclosures? |
| **74** | **Nv18.2**  **Nv20.4** | Is there a protocol for cleaning and disinfection activities for each enclosure? |
| **75** | **Nv18.2**  **Nv20.4** | Are updated risk assessment sheets available for the disinfectants and detergents used? |
| **76** | **Nv18.3** | Is the staff adequately trained for their roles? |
| **77** | **Nv18.3** | If tasks require specific training and certifications (e.g., a license for operating mechanical equipment), are these certifications present and up-to-date? |
| **78** | **Nv20.1** | Is there a procedure in place to verify the parameters of the water in the tanks? |
| **79** | **Nv20.1** | Is there a predetermined frequency for checking the parameters of the water in the tanks? |
| **80** | **Nv20.2** | Is there a protocol for managing non-conformities in the analytical results of water quality monitoring in the tanks? |
| **81** | **Nv20.3** | Is there a register where all the analytical results of the water quality monitoring in the tanks are recorded? |
| **82** | **Nv24.1** | Is there a protocol for controlling pest animals? |
| **83** | **Nv24.1** | Is there a map showing the distribution of baits? |
| **84** | **Nv24.1** | Is there a log of trap checks with bait? |
| **85** | **Nv24.1** | Are updated safety and usage sheets available for the used products? |
| **86** | **Nv25.1** | Is there a protocol for the procurement of feed, its storage, and administration in compliance with hygiene and health standards? |
| **87** | **Nv25.2** | Are there dedicated premises for the storage of animal feed, separated by type, and maintained in suitable hygienic conditions? |
| **88** | **Nv25.2** | Is there a protocol for the storage of animal feed, separated by type, in suitable hygienic conditions? |
| **89** | **Nv25.3** | Is there a procedure for monitoring the proper storage of animal feed? |
| **90** | **Nv27.1** | Is there a protocol for managing any non-compliance with animal feed? |
| **91** | **Nv27.1** | Is there a protocol for managing any non-compliance with animal feed? |
| **92** | **Nv27.2** | Is there a protocol for the preparation of animal feed in compliance with hygiene and health standards? |
| **93** | **Nv27.3** | Is there a protocol that involves separating different types of feed during preparation to prevent cross-contamination? |
| **94** | **Nv27.3** | Does the protocol for feed preparation involve the separate use of utensils for processing meat and vegetables, ensuring the absence of cross-contamination? |
| **95** | **Nv29.1** | Is there a procedure to verify compliance with hygiene standards during the transport and administration of feed? |
| **96** | **Nv31.1** | Is there a protocol establishing the procedures for isolation/quarantine of various species? |
| **97** | **Nv33.1** | Are there procedures in place to provide veterinary instructions for cleaning/disinfecting the isolation/quarantine areas and tanks to the assigned staff? |
| **98** | **Nv33.2** | Are there health protocols for isolation? |
| **99** | **Nv33.3** | Is there a list of products suitable for disinfection? |
| **100** | **Nv33.3** | Do the products used meet the veterinary recommendations for cleaning and disinfecting the enclosures and isolation/quarantine tanks? |
| **101** | **Nv33.4** | Is there a protocol for the cleaning and disinfection process of enclosures and isolation/quarantine tanks? |
| **102** | **Nv21.1** | Are the isolation protocols prepared following the competent Local Health Authority (ASL)? |
| **103** | **Nv21.2** | Are the cleaning and disinfection protocols monitored? |
| **104** | **Nv21.2** | Is there a person responsible for monitoring the application of cleaning and disinfection protocols? |
| **105** | **Nv34.1** | Is there a procedure for reporting any risks of infectious diseases transmitted by the personnel responsible for animals, as indicated by current regulations? |
| **106** | **Nv34.2** | Is there a record of personnel training regarding the correct use of personal protective equipment (PPE)? |
| **107** | **Nv34.2** | Are there preventive procedures in place to avoid the risk of transmission of infectious diseases? |
| **108** | **Nv34.2** | Is there someone responsible for ensuring the correct use of Personal Protective Equipment (PPE)? |
| **109** | **Nv34.2** | Is there a procedure for monitoring the correct use of Personal Protective Equipment (PPE)? |
| **110** | **Nv34.3**  **Nv36.2** | Is the assessment of zoonotic risk for personnel carried out with the company doctor? |
| **111** | **Nv35.1** | Is there a taxon-specific protocol for the management of infectious and parasitic diseases drafted by the veterinarian? |
| **112** | **Nv36.1** | Are prevention plans diversified based on species or taxon? |
| **113** | **Nv36.2** | Is there a protocol for the analysis of zoonotic disease risk? |
| **114** | **Nv36.2** | Is there a protocol for the prevention of zoonotic diseases? |
| **115** | **Nv37.1** | Are corrective and preventive actions taken in case of zoonotic risk? |
| **116** | **Nv37.1** | Are corrective and preventive actions taken in the event of zoonotic transmission? |
| **117** | **Nv37.2** | Is there a management protocol containing corrective and preventive actions in case of zoonotic transmission? |
| **118** | **Nv37.3** | Is the staff subjected to regular medical check-ups? |
| **119** | **Nv37.3** | Has a zoonotic risk assessment been conducted with the company doctor for the staff? |
| **120** | **Nv37.3** | Do staff visits occur at predetermined intervals? |
| **121** | **Nv37.3** | Has the preventive medicine plan been co-signed by the responsible veterinarian and the company doctor? |
| **122** | **Nv39.1** | In case of animal death, are investigations and monitoring carried out? |
| **123** | **Nv39.1** | Is there a protocol for the disposal of animal carcasses? |
| **124** | **Nv40.1**  **Nv40.2** | Is there a necropsy room available? |
| **125** | **Nv40.1** | Are there a refrigerated cell and a freezer for deceased animals? |
| **126** | **Nv40.1** | Is there a room for conducting post-mortem examinations? |
| **127** | **Nv40.1** | If post-mortem examinations are conducted in external facilities (e.g., Istituti Zooprofilattici Sperimentali or universities), is there a protocol for safe transportation? |
| **128** | **Nv40.2** | Are the specific rooms for necropsies washable and disinfectable? |
| **129** | **Nv40.2** | Are requirements for the rooms used for carrying out necropsies adopted and respected? |
| **130** | **Nv41.1** | Are methods of marking and identifying individual animals adopted, including through individual photography? |
| **131** | **Nv41.2**  **Nv41.6** | Is there a protocol for the registration and identification of hosted animals? |
| **132** | **Nv41.2**  **Nv41.5** | Is there a complete and updated register, whether computerized or paper-based, of the individuals present, including identifiers where obligatory or possible? |
| **133** | **Nv41.3** | Is there a protocol in place to evaluate the effectiveness of individual identification operations for the various species? |
| **134** | **Nv41.3** | Is there an identification marking for all animals? |
| **135** | **Nv41.4** | Are the animals registered in a national database? |
| **136** | **Nv41.6** | Does the registration of individual subjects in the register (entry/exit or intake/release) include the indication of the marking method, the marking details, and the individual identification code? |
| **137** | **Nv42.1** | Is there a protocol for identifying groups of animals? |
| **138** | **Nv41.2** | Are there transport and entry documents for the animals? |
| **139** | **Nv55.1** | Do the veterinarians working in the facility have documented experience in wildlife and zoo animal medicine? |
| **140** | **Nv55.2** | Are there standards to evaluate the qualifications and titles required for the veterinarian working within the facility? |
| **141** | **Nv56.1** | Is the certified experience (scientific publications, college, specialization, master's degree) of the veterinarian or pathologist who performs necropsies verified? |
| **142** | **Nv58.1** | Is there a person responsible for preparing, facilitating the execution, and subsequent monitoring of the training and updating of personnel on internal procedures? |
| **143** | **Nv58.2** | Are the procedures accessible and easily consultable by all staff? |
| **144** | **Nv58.2** | Is there a methodology for disseminating, informing, and training all staff regarding the procedures? |
| **145** | **Nv58.3** | Is there a register of training and updates for staff on internal procedures? |
| **146** | **Nv59.1**  **Nv59.2** | Is the level of training/updating of personnel regarding animal health and welfare monitoring assessed? |
| **147** | **Nv59.1** | Is the level of training/updating taken into consideration in the selection of personnel following the level of responsibility within the structure? |
| **148** | **Nv59.3** | Are regular update meetings held with attendance records? |
| **149** | **Nv59.3** | Is staff training/updating path regarding quarantine/isolation procedures monitored? |
| **150** | **Nv60.1** | Are there criteria applied to identify individuals suitable to work in quarantine/isolation? |
| **151** | **Nv68.1** | Is specific training on behavioural monitoring and the repertoire of cared-for species verified? |
| **152** | **Nv68.2** | Are there criteria to evaluate the level of training/update of staff regarding behavioural monitoring? |
| **153** | **Nv68.2** | Is staff trained to monitor species-specific behaviours? |
| **154** | **Nv68.3** | Is behavioural monitoring conducted? |
| **155** | **Nv68.3** | Is there a protocol to monitor behavioural alterations in the various cared-for species? |
| **156** | **Nv68.4**  **Nv69.3** | Is there a program for providing environmental enrichment? |
| **157** | **Nv68.4** | Are there programs in place to ensure animal welfare? |
| **158** | **Nv69.1** | Is the staff trained in enrichment practices? |
| **159** | **Nv69.1** | Is there an enrichment coordinator? |
| **160** | **Nv69.1** | Is there a training program for staff on the design and use of enrichments? |
| **161** | **Nv69.2** | Are the environmental enrichments suitable for the different species? |
| **162** | **Nv69.2** | Are natural behaviours stimulated in various animal species through actions in the housing environment? |
| **163** | **Nv69.2** | Are there strategies implemented to ensure natural behaviours of the species according to their ethogram? |
| **164** | **Nv69.3** | Is there a program to verify the implementation and effectiveness of environmental enrichments? |
| **165** | **Nv70.1** | Are there suitable tools available for monitoring environmental parameters? |
| **166** | **Nv70.1** | Is there a protocol for monitoring environmental parameters in compartments and tanks? |
| **167** | **Nv71.1** | Is there a protocol ensuring training and updating of the staff involved in training, animal-visitor interactions, and/or performances? |
| **168** | **Nv71.2** | Is there a protocol for evaluating the training and updating of staff involved in training, animal-visitor interactions, and/or performances? |
| **169** | **Nv71.2** | Is there a protocol for the training and updating of staff involved in handling and transporting animals? |
| **170** | **Nv61.1** | Are there procedures in place to promote the dissemination and continuous improvement of knowledge on animal welfare and health with other facilities? |

**Themes and indicators’ codes:**

Nv2 - Theme MONITORING THE HEALTH CONDITIONS OF THE ANIMALS ACCORDING TO THEIR SPECIES

***Indicators:***

1. ***Evaluation of nutrition and hydration status***
2. ***Daily recordings by keepers***
3. ***Visual examination of the condition and, where applicable, behaviour***
4. ***Monitoring of major organ functions***
5. ***Assessment of sensory status (normal responsiveness to natural or induced stimuli)***
6. ***Presence of adequately trained and sufficient keepers***
7. ***Veterinary intervention in case of abnormalities/alterations***
8. ***Existence of medical records***
9. ***Body weight monitoring***
10. ***Monitoring of regular food and water intake***
11. ***Recording of the number of checks throughout the day***
12. ***Periodic evaluation of ethograms***
13. ***Presence of normal faeces according to species***

Nv4 - Theme ROUTINE EXAMS, INCLUDING PARASITOLOGICAL ONES

***Indicators:***

1. ***Performance of clinical examinations and periodic screening as required by law according to the species***
2. ***Conducting routine necropsies and histological examinations on deceased animals***
3. ***Implementation of species-specific prophylaxis based on epidemiologically relevant diseases in the area***

Nv7 - Theme REGULAR IMPLEMENTATION OF PREVENTIVE MEDICINE INTERVENTIONS

***Indicators:***

1. ***Evidence of the presence of a preventive medicine plan, including vaccinations against species-specific diseases***
2. ***Availability of veterinary medical records for each individual***
3. ***Presence of a veterinarian to perform interventions in the species***

Nv8 - Theme GARANZIA DI ASSISTENZA VETERINARIA DI ROUTINE

***Indicators:***

1. ***Presence of a designated individual and an active contract with fixed or affiliated veterinary personnel following Legislative Decree 73/2005 as required by law***
2. ***Presence of at least one resident veterinarian capable of assisting in case of emergencies***

Nv9 - Theme ROUND-THE-CLOCK VETERINARY ASSISTANCE AVAILABLE THROUGHOUT THE ENTIRE WEEK

***Indicators:***

1. ***Availability of equipped rooms and veterinary equipment in the facility as required by law***

Nv10 - Theme IMMEDIATE CARE AND ATTENTION BY THE VETERINARIAN FOR ANY ANIMAL IN CONDITIONS OF STRESS, ILLNESS, OR INJURY

***Indicators:***

1. ***Presence of individual clinical records demonstrating intervention, diagnosis, therapy, and reassessment of subjects after reporting***

Nv11 - Theme ADEQUATE AVAILABILITY OF TRANQUILIZERS AND ANESTHETICS IN THE FACILITY

***Indicators:***

1. ***Management of the pharmaceutical cabinet, also in digital mode.***
2. ***Assessment of the presence of drug registers, drug stock, narcotic registers, and order registers.***
3. ***Availability of equipment for the administration of anaesthetics or sedatives and for the eventual recovery of animals waking up from anaesthesia.***
4. ***Evaluation of authorizations issued by the competent Local Health Authorities (ASL).***

Nv13 - Theme CORRECT HANDLING OF ANIMALS TO AVOID STRESS OR JEOPARDIZE THE ANIMALS' GOOD PHYSICAL AND PSYCHOLOGICAL CONDITION

***Indicators:***

1. ***Presence of protocols and enclosures for handling and immobilizing animals as required by regulations.***

Nv14 - Theme TRAINING OF THE STAFF RESPONSIBLE FOR THE ANIMALS TO IMMEDIATELY COMMUNICATE ANY IMPEDIMENTS IN CARRYING OUT THEIR DUTIES TO THE MANAGEMENT

***Indicators:***

1. ***Presence of a procedure to report impediments in carrying out one's duties.***
2. ***Training courses provided by the company for each staff member.***

Nv64 - Theme ADEQUATE STRUCTURES AND EQUIPMENT FOR THE RECOVERY AND CONTAINMENT OF ANIMALS WITHIN THE ZOOLOGICAL FACILITY

***Indicators:***

1. ***Presence of adequate structures for containment and transportation of animals (fences of sufficient height, double gates, electrified fencing, moats, etc.) species-specific as required by law.***

Nv18 - Theme ADEQUATE CLEANING OF THE ENCLOSURES

***Indicatore:***

1. ***Periodic visual assessments are conducted by specifically designated staff members.***
2. ***Operational sheet with a schedule of cleaning and disinfection activities for each department***
3. ***Verification of tools and equipment and use by personnel with the relevant licenses for mechanical means***

Nv20 - Theme REGULAR MONITORING OF WATER QUALITY IN THE TANKS

***Indicators:***

1. ***Conducting certified physical-chemical and bacteriological analyses at regular intervals or in case of changes in the water supply source or maintenance of the system***
2. ***Presence of appropriate analytical results following water monitoring and use of reference parameters***
3. ***Availability of control logs***
4. ***Presence of cleaning protocols and frequency***

Nv24 - Theme EFFECTIVE AND SECURE PROGRAM FOR CONTROLLING INVASIVE SPECIES

***Indicators:***

1. ***Presence of actions aimed at controlling pests (rodent control plans, description of active ingredients used, recording of treatments, etc.) as indicated in D.Lgs 73/2005***

Nv25 - Theme COMPLIANCE FOR HYGIENE STANDARDS IN THE STORAGE OF FOOD AND BEVERAGES FOR ANIMALS

***Indicators:***

1. ***Presence of a protocol for managing feed according to hygiene standards***
2. ***Presence of dedicated premises for the storage of food and feed, separated by type, in suitable hygienic conditions***
3. ***Periodic control sheet signed by the facility's manager or their delegate***

Nv27 - Theme COMPLIANCE FOR HYGIENE STANDARDS IN FOOD AND BEVERAGES PREPARATION

***Indicators:***

1. ***Assessment and management of non-conformities***
2. ***Operating manual and control sheet approved by the responsible person of the facility or their delegate***
3. ***Procedures applied to avoid cross-contamination***

Nv29 - Theme ADHERENCE TO HYGIENE STANDARDS IN FOOD AND BEVERAGE ADMINISTRATION

***Indicators:***

1. ***Cleanliness of containers used for transporting food and beverages check, as well as the containers in which they are administered***

Nv31 - Theme QUARANTINE/ISOLATION SPACES COMPLIANT WITH THE NEEDS AND CHARACTERISTICS OF THE INDIVIDUALS PRESENT WITHIN THEM

***Indicators:***

1. ***Presence of taxa-specific isolation and quarantine management protocol/procedures***

Nv33 - Theme ACQUISITION AND IMPLEMENTATION OF VETERINARY INSTRUCTIONS FOR CLEANING AND DISINFECTION OF ENCLOSURES AND ISOLATION/QUARANTINE TANKS

***Indicators:***

1. ***Staff training***
2. ***Presence of written instructions for each quarantine/isolation conducted (sampling control)***
3. ***Disinfection of premises with appropriate products chosen according to the species and the presence of animals***
4. ***Cleaning and disinfection register***

Nv21 - Theme PROPER CLEANING AND DISINFECTION OF ISOLATION OR QUARANTINE ENCLOSURES AND TANKS

***Indicators:***

1. ***Agreed management with the competent veterinary service***
2. ***Written protocols check***

Nv34 - Theme TRAINING OF THE STAFF HANDLING THE ANIMALS TO IMMEDIATELY COMMUNICATE TO THE MANAGEMENT ANY CONTAGION OR RISKY CONTACTS FOR DISEASES TRANSMISSIBLE TO THE ANIMALS.

***Indicators:***

1. ***Evidence of a procedure for the immediate reporting of any contagious infectious diseases among animals as required by current regulations.***
2. ***Verification of the use of Personal Protective Equipment (PPE) and coordination with the company doctor.***

Nv35 - Theme PLAN FOR THE MANAGEMENT OF INFECTIOUS AND PARASITIC DISEASES, INCLUDING CORRECTIVE AND PREVENTIVE ACTIONS

***Indicators:***

1. ***Presence of a protocol for the management of infectious and parasitic diseases drafted by the veterinarian with adequate taxa-specific management procedures***

Nv36 - Theme ZOONOSIS RISK ASSESSMENT

***Indicators:***

1. ***Presence of a preventive medicine plan for various species aimed at preventing zoonoses***
2. ***Presence of a risk analysis plan as required by law***

Nv37 - Theme CORRECTIVE AND PREVENTIVE ACTIONS TAKEN IN CASE OF ZOONOSIS

***Indicators:***

1. ***Evaluation of the degree of collaboration with competent authorities***
2. ***Presence of a document indicating preventive and corrective actions***
3. ***Periodic medical check-ups for staff***

Nv39 - Theme IDENTIFICATION OF THE CAUSES OF ANIMAL DEATH THROUGH POST-MORTEM INVESTIGATIONS

***Indicators:***

1. ***Presence of a protocol for the management of deceased animals including adequate investigation, monitoring, and disposal.***

Nv40 - Theme CLEANING AND DISINFECTION OF THE PREMISES USED FOR NECROPSY EXAMINATIONS

***Indicators:***

1. ***Presence of a necropsy room and dedicated freezers.***
2. ***Premises must be washable and disinfectable.***

Nv41 - Theme SPECIMENS IDENTIFICATION

***Indicators:***

1. ***Use of appropriate equipment.***
2. ***Presence of a complete and updated register of individuals, with identifiers for each individual.***
3. ***Number of individually identified animals/total number of animals.***
4. ***Inclusion of animals in a national database.***
5. ***Presence of a computerized list of animals.***
6. ***Report the marking details on the animal loading/unloading registers.***

Nv42 - Theme GROUPS OF ANIMALS IDENTIFICATION

***Indicators:***

1. ***Clear description of the criteria adopted for group identification.***
2. ***Presence of transportation/entry documents.***

Nv55 - Theme EXPERIENCE AND SPECIFIC TRAINING OF THE VETERINARIAN WHO CARRIES OUT THEIR ACTIVITY WITHIN THE FACILITY

***Indicators:***

1. ***Analysis of curriculum vitae and study of obtained references and held titles.***
2. ***Assessment of field competencies by a third-party veterinarian with proven experience.***

Nv56 - Theme ANALYSIS OF CURRICULUM VITAE AND EXAMINATION OF OBTAINED REFERENCES AND QUALIFICATIONS. ASSESSMENT OF SPECIFIC TRAINING AND EXPERIENCE IN CONDUCTING NECROPSIES.

***Indicators:***

1. ***Certified experience (scientific publications, college degrees, specialization, master's degrees).***

Nv66 - Theme CONTINUOUS PROFESSIONAL DEVELOPMENT OF THE KEEPERS

***Indicators:***

1. ***Presence of a protocol indicating criteria for updating and dedicated resources.***

Nv58 - Theme TRAINING AND UPDATING OF STAFF ON INTERNAL PROCEDURES

***Indicators:***

1. ***Presence of clear internal procedures easily accessible to all staff.***
2. ***Evidence of dissemination of procedures to all staff.***
3. ***Records of internal meetings.***

Nv59 - Theme TRAINING AND UPDATING OF STAFF ON ANIMAL HEALTH, WELFARE, AND THEIR MONITORING.

***Indicators:***

1. ***Evaluation of obtained master's degrees and specializations.***
2. ***Assessment of the curriculum vitae and professional experience. Evidence of staff participation in refresher courses on animal health, welfare, and monitoring. Evaluation of the educational impact on staff through questionnaires and practical tests.***
3. ***Holding regular meetings with attendance records.***

Nv60 - Theme SPECIFIC TRAINING FOR STAFF HANDLING ANIMALS IN QUARANTINE/ISOLATION

***Indicators:***

1. ***Register of authorized staff to work in quarantine/isolation.***

Nv68 - Theme SPECIFIC TRAINING ON BEHAVIOURAL MONITORING AND SPECIES ETHOLOGICAL REPERTOIRE FOR THE CARED SPECIES

***Indicators:***

1. ***Evaluation of specialization degrees/masters/certified training***
2. ***Evaluation of curriculum vitae - Training of staff on behavioural monitoring and repertoire of various species***
3. ***Periodic evaluation of behavioural monitoring***
4. ***Presence of environmental enrichments***

Nv69 - Theme TRAINING ON THE DESIGN AND USE OF ENRICHMENTS

***Indicators:***

1. ***Staff training program***
2. ***Presence of suitable environmental enrichments tailored to different species, correctly positioned and potentially replaced periodically***
3. ***Presence of written protocols for individual species***

Nv70 - Theme TRAINING ON MONITORING ENVIRONMENTAL PARAMETERS IN ENCLOSURES AND TANKS

***Indicators:***

1. ***Verification of the presence of adequate instrumentation***

Nv71 - Theme SPECIFIC TRAINING FOR STAFF RESPONSIBLE FOR TRAINING, ANIMAL-VISITOR INTERACTIONS, AND/OR EXHIBITIONS

***Indicators:***

1. ***Staff training program***
2. ***- Attendance register for staff training sessions - Training program for staff on animal handling and transportation procedures***

Nv61 - Theme COLLABORATION AND EXCHANGE WITH OTHER FACILITIES ON ANIMAL WELFARE AND HEALTH DATA

***Indicators:***

1. ***Sharing and exchanging data and observations on animal welfare and health through partnerships with other zoos and research institutions.***
